# Intra and Inter-Spore Variability in *Rhizophagus irregularis AOX* Gene

**DOI:** 10.1371/journal.pone.0142339

**Published:** 2015-11-05

**Authors:** Catarina Campos, Hélia Cardoso, Amaia Nogales, Jan Svensson, Juan Antonio Lopez-Ráez, María José Pozo, Tânia Nobre, Carolin Schneider, Birgit Arnholdt-Schmitt

**Affiliations:** 1 EU Marie Curie Chair, ICAAM—Instituto de Ciências Agrárias e Ambientais Mediterrânicas, IIFA-Instituto de Formação e Investigação Avançada, Universidade de Évora, Núcleo da Mitra, Évora, Portugal; 2 Nordic Genetic Resource Center, Alnarp, Sweden; 3 Department of Soil Microbiology and Symbiotic Systems, Estación Experimental del Zaidín-Consejo Superior de Investigaciones Científicas (EEZ-CSIC), Granada, Spain; 4 Inoq GmbH, Solkau, Schnega, Germany; The University of Wisconsin—Madison, UNITED STATES

## Abstract

Arbuscular mycorrhizal fungi (AMF) are root-inhabiting fungi that form mutualistic symbioses with their host plants. AMF symbiosis improves nutrient uptake and buffers the plant against a diversity of stresses. *Rhizophagus irregularis* is one of the most widespread AMF species in the world, and its application in agricultural systems for yield improvement has increased over the last years. Still, from the inoculum production perspective, a lack of consistency of inoculum quality is referred to, which partially may be due to a high genetic variability of the fungus. The alternative oxidase (AOX) is an enzyme of the alternative respiratory chain already described in different taxa, including various fungi, which decreases the damage caused by oxidative stress. Nevertheless, virtually nothing is known on the involvement of AMF AOX on symbiosis establishment, as well on the existence of *AOX* variability that could affect AMF effectiveness and consequently plant performance. Here, we report the isolation and characterisation of the *AOX* gene of *R*. *irregularis* (*RiAOX*), and show that it is highly expressed during early phases of the symbiosis with plant roots. Phylogenetic analysis clustered *RiAOX* sequence with ancient fungi, and multiple sequence alignment revealed the lack of several regulatory motifs which are present in plant AOX. The analysis of *RiAOX* polymorphisms in single spores of three different isolates showed a reduced variability in one spore relatively to a group of spores. A high number of polymorphisms occurred in introns; nevertheless, some putative amino acid changes resulting from non-synonymous variants were found, offering a basis for selective pressure to occur within the populations. Given the *AOX* relatedness with stress responses, differences in gene variants amongst *R*. *irregularis* isolates are likely to be related with its origin and environmental constraints and might have a potential impact on inoculum production.

## Introduction

The interaction of arbuscular endomycorrhizal fungi (AMF) (phylum Glomeromycota) and terrestrial plants is a highly ancient (~ 450 million years) and successful symbiosis maintained by the vast majority of plant species, including angiosperms, gymnosperms and some genus of bryophytes and pteridophytes [1,2]. AMF are obligate biotrophs and exist in the soil as extraradical mycelium, sporocarps and spores; following spore germination, the hyphal germ tube grows through the soil in search of a host root. Once contact between the symbionts is established, the fungus forms an appressorium on the root surface via which it enters the root forming highly branched structures (arbuscules) inside root cortical cells [2,3]. These arbuscules are connected to an extensive network of extra-radical mycelium providing multifunctional actions to the plant, from nutrient uptake to alleviation against a diversity of stresses including soil metal toxicity [4]. In return, the fungus acquires carbon from the plant, via the intraradical mycelium [2].

Due to the beneficial effects that AMF can have on plant performance, the interest on AMF application in agricultural systems has been increasing [5]. Nevertheless, it is not a widely accepted strategy and some mistrust is present within farmers as they often observe inconsistency of results in crop perform enhancement upon AMF application [6]. Also, companies dedicated to AMF inoculum production have been reporting a high heterogeneity of inocula quality from year to year. Whether several reasons can be behind this inconsistency (e.g. genetics, behavioural, ecologic), it is crucial to develop strategies that could tackle it. As a first step, at the level of inoculum production, tools that could allow maintaining and assessing the quality and uniformity of the inoculum would be of high interest. AMF present high genetic diversity which can contribute for unstable product quality of different AMF isolates [7]. AMF spores contain typically hundreds of nuclei [8] and can carry several genetic variants [9]. In this sense, the development of a marker, focused on a selected gene, directly linking a sequence motif (identified at genomic level) to phenotypic variation (inoculum quality) could represent a valid approach to be pursued.

The starting point of marker development through candidate gene approach is the knowledge of a gene with an assigned function. Following, it is required the existence of variability among allele sequences from which polymorphic, functional motifs affecting phenotype can be identified. Here, there are presented these two phases of functional marker development based on a hypothesis-driven approach previously presented by Vicente and co-workers [10] which focused on the alternative oxidase (*AOX*) as the target gene. According to that hypothesis, *AOX* could be a marker to characterise biodiversity/gene diversity and at the same time the functional integrity of AMF communities. AOX is a key enzyme in the alternative respiratory chain described in plants, fungi and also in some animals [11], that reportedly either decreases the damage caused by oxidative stress or minimises the production of reactive oxygen species (ROS) generation [12,13]. Research with plants confirmed that AOX fulfils physiological functions in growth, developmental events and in response to environmental stresses such as cold, drought, heat or nutrient deficiency (see review of Vanlerberghe (2013) [14]). For these reasons, *AOX* has been pointed out as a target gene for functional marker development related with plant plasticity [15,16]. Studies on fungi *AOX* are much more scarce; nevertheless, *AOX* was already isolated in several fungi species, and for instance has been related with defence against exogenous oxidative stress [17–19] or with the regulation of the biotrophic development and transition to necrotrophic stage in pathogenic fungi [20].

Tamasloukht et al. [21] showed that the respiratory activity of AMF is stimulated by root exudates, since an increase in oxygen consumption was noted after approximately 90 min of exposure. It was also observed that this stimulation of fungal respiration preceded an intense hyphal branching [21]. Therefore, these authors have speculated on the role of alternative respiration during the presymbiotic phase as enabling survival of AMF spores, being AOX the key enzyme in that pathway. More recently, Besserer and colleagues [22] further published important results showing the involvement of AOX activity in AMF as response upon the plant signalling exudate strigolactone.

In the present work, we report the isolation and characterisation of the *AOX* gene in the AMF *Rhizophagus irregularis*, the most AMF used as inoculant in agriculture, and we also show the involvement of *RiAOX* on symbiosis establishment by measuring the transcript accumulation over time at both host plant and AMF. The existence of *RiAOX* gene variability among different isolates of *R*. *irregularis* was further investigated by high throughput sequencing, considering the future development of functional markers to assess AMF inoculum quality.

## Material and Methods

### 2.1. Isolation and characterisation of the *RiAOX* gene sequence

#### 2.1.1. *RiAOX* complete gene isolation

*R*. *irregularis* single spores, collected from the *in vivo* inoculum INOQ69 provided by INOQ GmbH company (Solkau, Schnega, Germany) (http://inoq.de), were directly crashed in Whatman FTA paper (GE Healthcare, Little Chalfont, England) and immediately used for total DNA amplification using the REPLI-g Mini Kit (Qiagen, Hilden, Germany). A first approach to amplify the gene was based on the design of primers located at the 5’ and 3’ -UTRs in order to isolate the full-length sequence (based on the sequence available at NCBI, acc. no. GW102706). However, due to the inefficiency of that strategy, a new set of primers located within the ORF was designed for amplification of a fragment with a size of ca. 700 bp (RiFw_80: 5’-CATTTACTATCCACGCATCACGA-3’ and RiRev_651: 5’-TCCAACCACACGATGAGC-3’). PCR was conducted with Ready-To-Go PCR Beads (GE Healthcare, Little Chalfont, England) using 3 μl of the amplified DNA (previously diluted 1:20) as template and 0.2 μM of each forward and reverse specific primers. PCR was carried out for 35 cycles in a 2720 Thermocycler (Applied Biosystems, Foster City, CA, USA), consisting each cycle in 94°C for 30 s, 50°C for 30 s and 72°C for 1 min. An initial step of 94°C for 5 min and a final step of 72°C for 10 min were performed.

In order to isolate the 5’ and 3’ -UTRs, total RNA was extracted from 1 cm^3^ of an *in vitro* culture of *R*. *irregularis* strain INOQ69, which included spores and mycelium. Total RNA was extracted using the RNeasy ® Micro Kit (Qiagen, Hilden, Germany) following manufacturer’s protocol. RNA purity and concentration were assessed in a NanoDrop-2000C spectrophotometer (Thermo Scientific, Wilmington, DE, USA). Ten ng of total RNA were used as template for cDNA synthesis using the SMARTer^TM^ RACE cDNA Amplification Kit (Clontech, Mountain View, USA). For 5’and 3’ -UTRs isolation, a reverse and a forward specific primers were designed (GSP2: 5’- GAAACTGTTGCCGCAGTTCCTGGTATG-3’ and GSP1a: 5’- GCGTTCCCACCAGGTAGGTTGTGAAAT-3’, respectively) to be used in combination with a universal primer provided by the manufacturer. PCR was performed according to the protocol of the RACE cDNA Amplification Kit using the Advantage^®^ 2 PCR Kit (Clontech, Mountain View, USA).

For complete gene isolation, a forward and a reverse primer set were designed in the previously obtained 5’ and 3’ -UTRs regions (RiORF105Fw: 5’-AATGATTCGCTCGGTTTTTC-3’ and RiORF1137Rev: 5’-TTATTTACTCCATTTCCATTTTTC-3’). Total DNA of a single *in vitro* spore of *R*. *irregularis* strain INOQ69 was amplified as described above and 1 μl was directly used as template for PCR using 0.2 μM of each specific primer. The enzyme Phusion™ High-Fidelity DNA Polymerase (Thermo Fisher Scientific, Waltham, MA, USA) was used according to the manufacturer’s protocol. The amplification consisted in 35 cycles of 98°C for 10 s, 50°C for 20 s and 72°C for 1 min. An initial step of 98°C for 2 min and a final step at 72°C for 10 min were performed. The obtained amplicons were cloned onto a pGEM^®^ –T Easy vector (Promega, Madison, Wisconsin, USA) and used for transformation of *E*. *coli* JM109 (Promega, Madison, WI, USA) competent cells. Selected recombinant clones were send for commercial sequencing (Macrogen, Seoul, Korea: www.macrogen.com) using T7 and SP6 primers (Promega, Madison, WI, USA).

#### 2.1.2. *RiAOX* sequence analysis

CLC Genomics workbench 7.5.1 platform (CLCbio, Aarhus N, Denmark) was used to edit the isolated *AOX* sequences. Homology of obtained sequences was confirmed in NCBI GenBank database (National Center for Biotechnology Information, Bethesda, MD, http://www.ncbi.nlm.nih.gov/) using BLASTn and BLASTp algorithm [23].

The exon-intron structure and the location of the AOX (Ferritin-like super family) conserved domain was compared between *R*. *irregularis* and other fungal species using the Fancy Gene v1.4 software (freely available at http://bio.ieo.eu/fancygene/). The genomic *AOX* sequences of *Rozella allomycis*, *Agaricus bisporus*, *Laccaria bicolor*, *Macrophomina phaseolina*, *Cryptococcus neoformans*, *Coccidioides posadasii*, *Neurospora crassa*, *Lichtheimia corymbifera*, *Candida albicans*, *Talaromyces stipitatus* and *Spraguea lophii* were retrieved from EMBL-EBI (http://www.ebi.ac.uk/) or NCBI (http://www.ncbi.nlm.nih.gov/).

The prediction of the fungal AOX N-termini that can support mitochondrial targeting sequence was performed on the translated peptide sequence of *R*. *irregularis* AOX and on other fungal AOX sequences using MITOPROT software (http://ihg.gsf.de/ihg/mitoprot.html).

A phylogenetic analysis of AOX proteins from *R*. *irregularis* and from other fungi and plant species was performed. AOX protein sequences from plant Monocots, Eudicots and Gymnosperm species were retrieved from PLAZA (http://bioinformatics.psb.ugent.be/plaza/), UniProt (http://www.uniprot.org/), Ensembl Plants (http://plants.ensembl.org/index.html) and/or NCBI (see accessions on S1 List). Fungi AOX sequences were retrieved from EMBL-EBI, UniProt and/or NCBI (accessions on S1 List). Sequence alignments were performed on MAFFT (http://mafft.cbrc.jp/alignment/server/) under the default parameters and a selection of best-fit models of amino acid (aa) replacement was performed using ProtTest 2.4 (http://darwin.uvigo.es/software/prottest2_server.html). Using the selected best-fit model (LG+I+G) a Maximum Likelihood phylogenetic analysis was performed on MEGA V6.0 [24], with 150 bootstraps. Two bacterial AOX sequences were used as outgroup (*Rhodanobacter* sp. and *Afipia felis*).

To identify distinctive features of fungi and plant AOX proteins, multiple alignments were performed using the CLC software on the helices *α*2, *α*3, *α*5 and *α*6, which form the four-helix bundle accommodating the diiron center, on the helices *α*1 and *α*4, which form the hydrophobic region on the AOX molecular surface, and on region 3, previously defined as possibly important for plant AOX activity [25]. The regions involving the two conserved cysteine residues (CysI and CysII) which in plants are responsible for the *α*-keto acid regulation were also compared between plant and fungal sequences.

### 2.2. Study of *AOX* transcript variation during symbiosis establishment on AMF and host plant

#### 2.2.1. Experimental design and sample collection

To study the involvement of AOX at transcriptional level on both AMF and host plant during mycorrhizal colonisation, an experimental trial was set up using tomato (*Solanum lycopersicum* L. cv. MoneyMaker), which is often selected as model plant for experiments with AMF, and *R*. *irregularis* (BEG 121 isolate) (former *Glomus intraradices*).

Tomato seeds were surfaced-sterilised in 4% sodium hypochlorite containing 0.02% (v/v) Tween 20, rinsed thoroughly with sterile water and germinated for 2 days in a plate on moistened filter paper at 25°C in darkness. Subsequently, tomato seedlings were grown hydroponically in 3 L plastic containers with Long Ashton nutrient solution and at constant aeration. The nutrient solution was replaced once a week. After 2 weeks, plantlets were individually transferred to 0.45 L pots with sterile sand:soil:vermiculite (3:1:1) mixture (soil had a pH of 7.2, 1.6% organic matter, 2.1 μg Kg^-1^ of N, 1.7 μg Kg^-1^ of P and 0.8 μg Kg^-1^ of K as nutrient concentration).

Half of the plants (24 plants) were inoculated by adding 10% (v:v) of *R*. *irregularis* inoculum, which consisted in *R*. *irregularis* kept in a soil-sand mixture containing extradical mycelium and spores, and mycorrhizal root fragments of *Trifolium repens* L. and *Sorghum vulgare*. This AMF had been isolated from a Mediterranean agricultural soil. The same amount of soil:sand mix, but free of AMF, was added to control non- mycorrhizal plants. Non-mycorrhizal plants received an aliquot of AM inoculum filtrated (< 20 μm) (meaning without propagules) to homogenize the microbial populations. Plants were randomly distributed in a greenhouse and grown at 24/16°C with 16 h photoperiod and 70% humidity, watered three times a week with Long Ashton nutrient solution [26] containing 25% of the standard P concentration.

Plants were harvested at different time points: 1, 2, 4 and 6 weeks post inoculation. Three plants (biological replicates) were collected per time point for both inoculated and non-inoculated plants (control). Samples taken from the root system were immediately frozen in liquid nitrogen and stored at -80°C for further analysis. A sample of each individual root system was also taken for mycorrhizal quantification through microscopy observation.

#### 2.2.2. Determination of mycorrhizal colonisation

Root samples of *R*. *irregularis* inoculated tomato plants were stained with trypan blue [27] and examined using a Nikon Eclipse 50i microscope and bright-field conditions. Root colonisation by the AMF was determined as previously described [28], using the MYCOCALC software (http://www.dijon.inra.fr/mychintec/Mycocal-prg/download.html). The parameters measured were the mycorrhizal colonisation frequency (*F %*) and intensity (*M %*), arbuscule (*A %*) and vesicle (*V %*) abundance, and the relative colonisation intensity (*m %*). At least five slides, containing 30 root pieces of 1 cm length, were analysed per biological replicate. Five replicates were analysed at each time point.

#### 2.2.3. RNA isolation and gene expression analysis by quantitative real time RT-PCR (qPCR)

Total RNA was extracted using Tri-Reagent (Sigma-Aldrich, MO, USA) according to manufacturer’s instructions. RNA was treated with RQ1 DNase (Promega, Wisconsin, USA), purified through a silica column using the NucleoSpin RNA Clean-up kit (Macherey-Nagel, PA, USA). Before storage at -80°C, RNA was quantified using a NanoDrop-2000C spectrophotometer (Thermo Scientific, Wilmington, DE, USA) and its integrity was checked by gel electrophoresis. First strand cDNA was synthesised with 1 μg of purified total RNA using the iScript cDNA Synthesis kit (Bio-Rad, CA, USA) according to the manufacturer’s instructions. Real time qPCR was performed using 1 μl of single stranded cDNA (diluted 1:10) and specific primers for the four tomato *AOX* genes (*SlAOX1a*, *SlAOX1b*, *SlAOX1c* and *SlAOX2*), and the *AOX* gene of *R*. *irregularis*. The tomato phosphate transporter *SlPT4*, usually used as molecular marker for arbuscular functionality with direct link with mycorrhiza development in the roots [29] was also evaluated (see primer details on S1 Table). Reactions were carried out in a iCycler iQ5 system (Bio-Rad) for 40 cycles, consisting each cycle in 94°C for 15 s, 55°C for 20 s and 72°C for 20 s. An initial step at 94°C for 2 min was considered. Three independent biological replicates were analysed per treatment. Three independent biological replicates were analysed per treatment and each sample was analysed in duplicate. The specificity of the different amplicons was checked by a melting curve analysis at the end of the amplification protocol.

Two genes for tomato (*SlEF-*1 and *SlActin2*) and two genes for *R*. *irregularis* (*RiTEF1a* and *RiBTub1*) were used as reference genes for further normalisation of the transcript data achieved on the target genes. Evaluation of expression stability for the reference genes was done using the statistical application *geNorm* [30]. Expression of target genes was evaluated by relative quantification using the geometric normalisation factors obtained from *geNorm*. Standard curves of a 4-fold dilution series from pooled cDNAs were used for PCR efficiency calculations [31].

Differences in the expression level of target genes between non-mycorrhizal and mycorrhizal roots were examined by a t-test, or when the data did not meet the normality and/or equal variance requirements with a Mann-Whitney Rank Sum test. Transcript levels between weeks post-inoculation were examined by a one-way analysis of variance (ANOVA) with Holm–Sidak *post-hoc* tests. When the data did not meet the normality and/or equal variance requirements, a Kruskal–Wallis one-way ANOVA on ranks with a Dunn’s test for *post-hoc* comparisons was performed instead. All expression data were analysed with the SigmaStat statistical package (Systat software, London, UK). Significance levels were set at *P* < 0.05.

### 2.3. Analysis of *RiAOX* sequence diversity at single spore level by high-throughput sequencing

#### 2.3.1. Isolate selection and DNA extraction at single spore level

Three single spores from three different *R*. *irregularis* isolates were used to investigate the *RiAOX* genomic sequence variability. The isolates used were BEG72 (provided by the Institute of Agrifood Research and Technology, IRTA, Spain), BEG144 (provided by Prof. Daniel Wipf, Agroécologie INRA-Université de Bourgogne, Dijon, France), and one isolate provided by INOQ GmbH, hereafter designated by INOQ isolate. Those AMF were, respectively, originally isolated in 1996 in Northeastern Spain [32], in 1979 in St. Petersburg, Russia (according to the International Bank for the Glomeromycota (http://www.i-beg.eu/)) and in 1996 in Breiningerberg/Aachen, Germany. All three inocula have been propagated on *in vivo* cultures ever since.

*In vivo* inoculum of BEG72 used in the experiment consisted in a mixture of mycorrhizal root fragments and spores on Terragreen® (Oil-Dri, UK) carrier substrate. The BEG144 and INOQ isolates used in this experiment consisted of *in vitro* AMF cultures (in carrot root organ cultures, ROC) established in 2013 from the original *in vivo* inoculum. Inoculum samples were observed under a binocular lens and spores were individually picked (three spores per isolate) with a pipette tip and placed in 0.2 ml tube containing 1 μl of sterile tap water. Spores were then crushed using a sterile pipette tip and the entire DNA content was amplified using the GenomiPhi V2 Whole-Genome Amplification Kit (GE Healthcare, Canada) according to manufacturers’ instructions. Synthesised DNA was stored at – 20°C until further use.

#### 2.3.2. Molecular identification of *R*. *irregularis*

To confirm the identity of all 9 individual spores as belonging to *R*. *irregularis*, the ribosomal rRNA sequences including the ITS-LSU-SSU regions [33], were amplified by PCR using the enzyme Phusion™ High-Fidelity DNA Polymerase (Thermo Fisher Scientific), cloned onto a pGEM^®^ –T Easy vector and send for commercially Sanger sequencing. Sequences from the *rRNA* gene of several *R*. *irregularis* and *R*. *intraradices* isolates together with *rRNA* sequences of *Glomus clarum* described in Stockinger et al. [34] and publically available in the NCBI database, were used to perform the clustering analysis with the sequences from the INOQ, BEG72 and BEG144 isolates. The region used for the analysis comprised ~1400 bp of the small subunit, ITS, and large subunit of the rRNA which is suitable to differentiate very closely related species such as *R*. *irregularis* and *R*. *intraradices* [35].

The multiple sequence alignment was performed using the CLUSTALW program in the software MEGA V6.0 [24]. Genetic distances were inferred by the Neighbour-Joining method [36] and the bootstrap consensus tree was inferred from 100 replicates.

#### 2.3.3. High throughput sequencing of *RiAOX* in single spores

In order to obtain enough amount of *RiAOX* amplicon in the 9 single spores regarding the high throughput sequencing using the Ion Torrent™ platform, it was necessary to follow a nested PCR approach.

Two sets of specific primers were used: Ri_F1: ATGATTCGCTCGGTTTTTC and Ri_R1: TTATTTACTCCATTTCCATTTTTC for first PCR, followed by a second PCR using the Ri_F2: CATAACACATTTCTTCGTAACACAAC and Ri_R2: CAGGAGCCTCATTAGTCAAACCG primers. The obtained amplicon had a size of 1209 bp. In both cases the reactions were conducted with the enzyme Phusion™ High-Fidelity DNA Polymerase (Thermo Fisher Scientific) for 35 cycles, consisting each cycle in 98°C for 10 s, 59°C/63°C (first/second PCR, respectively) for 20 s and 72°C for 1 min. An initial denaturation step at 98°C for 5 min and a final extension of 10 min at 72°C were also performed.

A minimum of 500 ng of PCR products were directly sent for sequencing using the Ion Torrent™ platform at STAB VIDA (http://www.stabvida.com/). The obtained FASTQ reads have been deposited in the Sequence Read Archive (SRA) from NCBI database.

Obtained reads were uploaded into the CLC Genomics workbench. The quality of sequencing reads was enhanced by trimming and discarding low quality sequences and only high quality FASTQ sequences were used for further analysis. Reads were mapped to a consensus reference sequence previously obtained from cloned Sanger sequences of *RiAOX* from the 3 isolates, using the following parameters: masking mode = no masking, mismatch cost = 2, insertion cost = 3, deletion cost = 3, length fraction = 0.5 and similarity fraction = 0.8, global alignment = no, non-specific match handling = map randomly, output mode = create read tracks, create report = yes, collect unmapped reads = no.

Once the reads were mapped to the reference, the mapped reads files were used as input to discover low frequency variants using quality score with the following parameters: ignore broken pairs = yes, minimum coverage = 10, minimum variant frequency (%) = 1, neighborhood radius = 5, minimum neighborhood quality = 15, minimum central quality = 20, remove pyro-error variants = yes, in homopolymer regions with minimum length = 3, with frequency below = 0.8, create track = yes, create annotated table = yes, create report = yes.

A comparison of all variants was then performed, being classified as Single Nucleotide Variation (SNV), Multiple Nucleotide Variation (MNV), Deletion (Del), Insertion (Ins) and Replacement (Repl). Comparison of all possible variants from the 9 spores was performed setting a frequency threshold (%) = 0. With this threshold it was possible to identify variants that are present in only one spore. On the contrary, to identify variants that are common to all 9 spores, a frequency threshold of 100% was set.

The frequency (defined as read count divided by read coverage) distribution of all variants, in relation to the total number of variants found for each spore was performed. The SNV frequency (read count divided by read coverage) along the gene sequence was performed for the three isolates.

The prediction of variants (SNVs and MNVs) that could putatively induce aa substitutions in the protein sequence was performed for each spore.

## Results

### 3.1. *RiAOX* sequence and structure analysis

**Fig 1:**
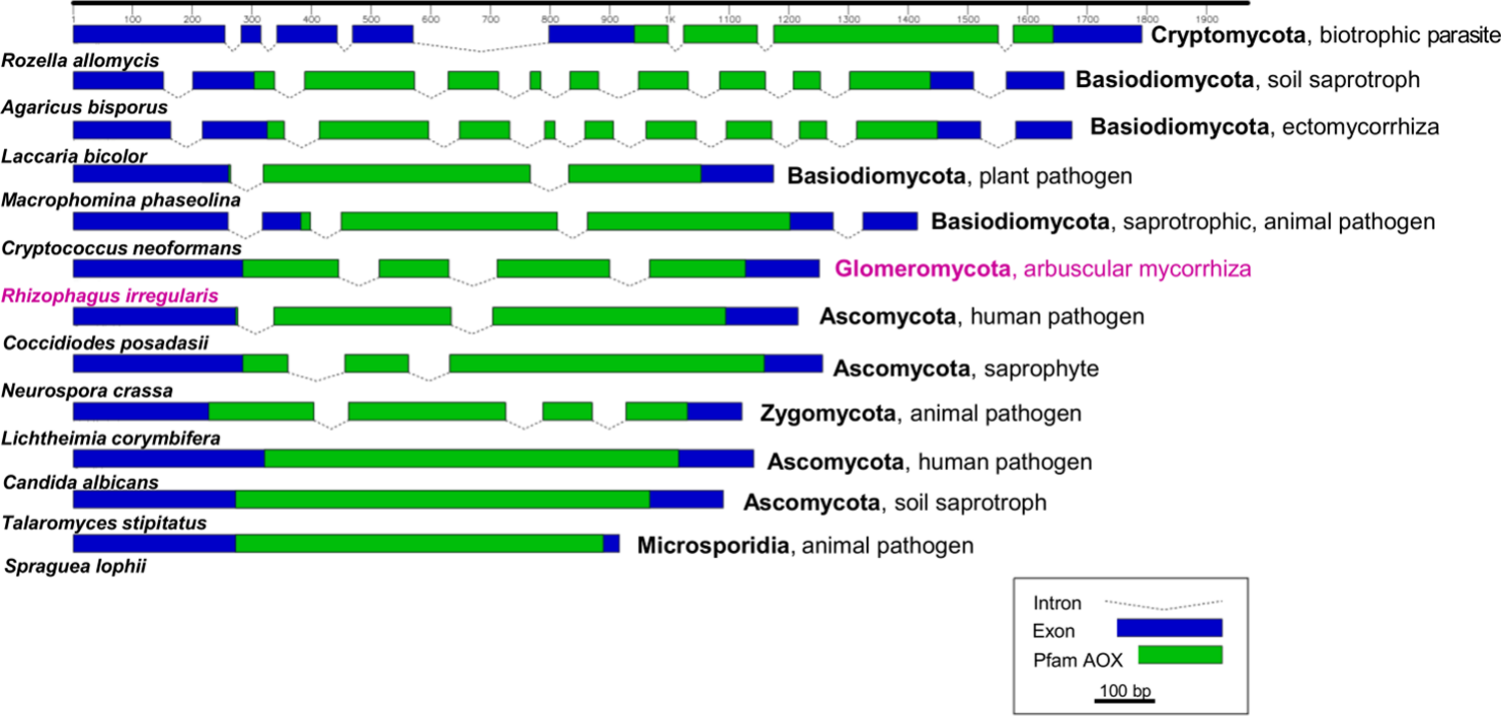
Comparison of exon-intron structure of *AOX* gene in *R*. *irregularis* and fungi from other phyla. Introns are represented by dashed lines and exons by blue rectangles. The green rectangles represent the location of the AOX encoded conserved domain, within the exons.

The full-length gene sequence of *R*. *irregularis AOX* has been deposited in GenBank (accession numbers KT423114 and KT423115). At the genomic level, *RiAOX* is composed by 4 exons interrupted by 3 introns, with a total size of 1250 bp (Fig 1). Variability among the exons and the introns within the *RiAOX* sequence is observed. The gene coding sequence, with 1032 bp and coding for a putative peptide of 343 aa, is composed by exon 1 (444 bp), exon 2 (116 bp), exon 3 (188 bp) and exon 4 (281 bp). The introns are considerably shorter, having 69, 81 and 68 bp for introns 1, 2 and 3, respectively.

The *AOX* genomic structure of *R*. *irregularis* was compared with those of other fungi (Fig 1). The structure of *AOX* gene varies considerably amongst fungal species and phyla: for instance, *T*. *stipitatus* (Ascomycota), *C*. *albicans* (Ascomycota) and *S*. *lophii* (Microsporidia) have no introns, whereas *R*. *allomycis* (Cryptomycota) has 7 introns *A*. *bisporus* (Basidiomycota) and *L*. *bicolor* (Basidiomycota) have 10 introns (Fig 1). Also, the location of the sequence encoding the conserved domain for AOX (Ferritin-like super family) differs between fungi: while many have it starting on exon 1, like *R*. *irregularis*, *L*. *corymbifera* (Zygomycota), *M*. *phaseolina* (Ascomycota), *N*. *crassa* (Ascomycota) and *C*. *posadasii* (Ascomycota), others such as *C*. *neoformans* (Basidiomycota) and *A*. *bisporus* have it starting on exon 2. *R*.*allomycis* differs from all the others since the AOX domain only starts on exon 5 (Fig 1).

**Fig 2:**
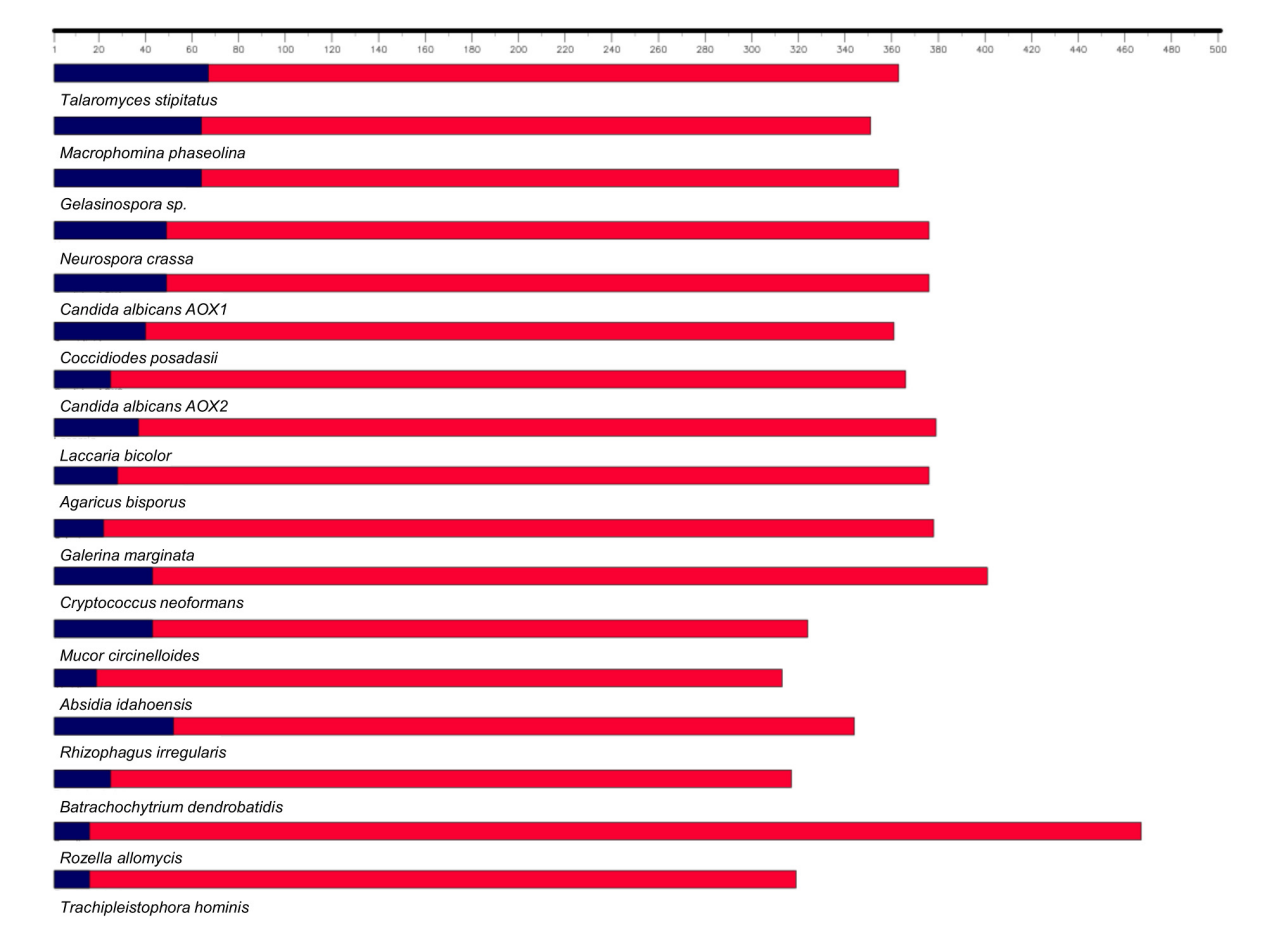
Predicted size of the N-terminal sequence that can support mitochondrial targeting and the cleavage site, in AOX proteins of different fungi. The N-terminal is indicated by blue rectangles.

The predicted N-terminal region of RiAOX protein that can support mitochondrial targeting sequence was determined as 52 bp length. Nevertheless, a great variation on the predicted mitochondrial targeting peptide was observed amongst fungi (Fig 2). The largest N-terminal was found in Ascomycota fungi (67 bp) and the smallest ones in *R*. *allomycis*, *B*. *dendrobatidis* and some Zygomycota species (12 bp). In some Zygomycota (*R*. *delemar* and *L*. *corymbifera*), Microsporidia (*S*. *lophii* and *N*. *parisii*) and Basidiomycota (*C*. *subvermispora*) the N-terminal was not predictable.

### 3.2. Phylogenetic analysis of plant and fungal AOX

**Fig 3:**
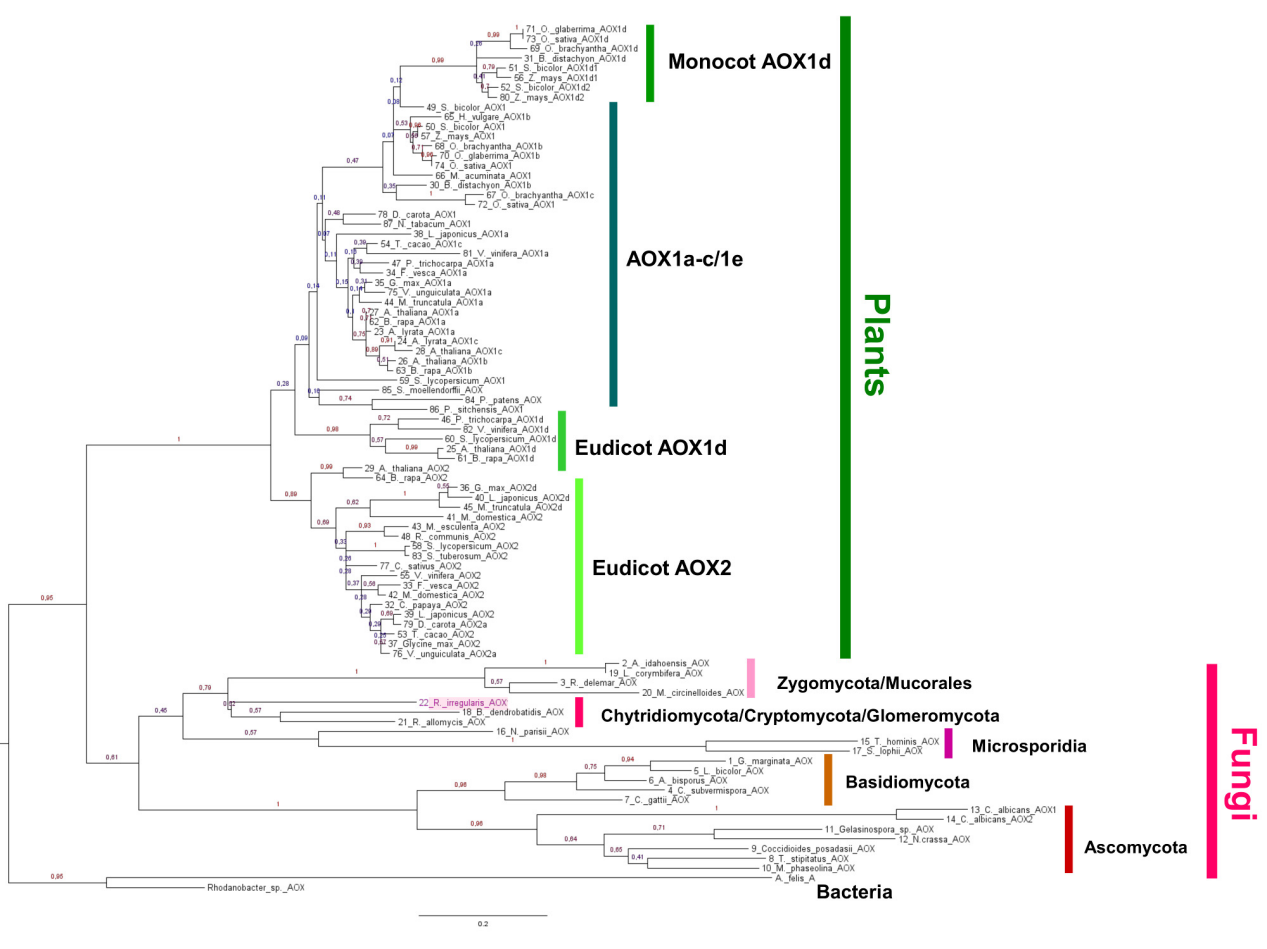
Maximum likelihood phylogenetic analysis of AOX protein sequences in fungi and plants. Two bacterial AOX sequences were placed as outgroup (*Rhodanobacter* sp. and *Afipia felis*). Location of *R*. *irregularis* is shaded. Fungal and plant AOX protein sequences were clearly separated by a ML bootstrap value of 0.95. The fungal sequences were grouped on two major clades, supported by a ML bootstrap value of 0.61: the Basidiomycota/Ascomycota clade, and a clade containing the Chytridiomycota, Cryptomycota, Glomeromycota, Microsporidia and Zygomycota phyla. Basidiomycota were separated from Ascomycota by a ML bootstrap of 1.

The phylogenetic analysis clearly differentiated between fungal and plant AOX protein sequences, which was supported by a high ML bootstrap value (0.95) (Fig 3). On the plant side, AOX sequences clustered into AOX2 and AOX1, and within the AOX1-subfamily the division in three main groups (AOX1a-c/1e, AOX1d from monocots, and AOX1d from eudicots) is evident, agreeing with the recent classification scheme proposed by Costa and co-workers [37]. However, while the monocot and eudicot AOX1d clusters were well supported by high bootstrap values, their positions relatively to the AOX1a-c/1e clade, as well as several positions inside this group were unresolved. Within the AOX2-subfamily, the AOX2d clade is also possible to identify, albeit with a lower branch support.

Fungal AOX sequences were grouped in two major clades: the Basidiomycota/Ascomycota clade, and another containing the Chytridiomycota, Cryptomycota, Glomeromycota, Microsporidia and Zygomycota phyla (Fig 3). Moreover, the Basidiomycota AOX sequences were completely separated from the Ascomycota.

*R*. *irregularis* AOX sequence clustered with the clade including *B*. *dendrobatidis* (Chytridiomycota) and with *R*. *allomycis* (Cryptomycota) (Fig 3). The sister group of *R*. *irregularis* clade comprised members of the Zygomycota (order Mucorales) and included *M*. *circinelloides*, *R*. *delemar*, *L*. *corymbifera* and *A*. *idahoensis*. The second closest clade to *R*. *irregularis* was composed by the Microsporidia members *Nematocida parisii*, *Trachipleistophora hominis* and *S*. *lophii*.

Multiple alignments of the helices *α*1 and *α*4 forming the hydrophobic region on the AOX molecular surface revealed that fungal and plant sequences are quite divergent N-terminally, especially regarding helix *α*1 and the region containing the plant conserved regulatory cysteine residue I (CysI) (S1 Fig). CysI is located N-terminal to *α*1. The helix *α*4 is fairly more conserved than *α*1. Some plant AOX lack the conserved CysI, having a serine residue instead (S1 Fig). The conserved CysI was not found in any of the fungal sequences.

The analysis of the region 3 [25] showed that whereas plant generally present the glutamic acid/aspartic acid, asparagine and valine (E/DNV) motif (the most evident exceptions go to AOX1d from monocots and some AOX1d from eudicots), the fungal sequences are much more divergent in this regard. The most similar to plant sequences were *R*. *allomycis* and *R*. *irregularis*. RiAOX showed the ENS motif, identical to that of some AOX1d from eudicots. *R*. *allomycis* presented the ENV motif, identical to most of the analysed plant species (S2 Fig).

The multiple alignments of the helices *α*2, *α*3, *α*5 and *α*6, which form the four-helix bundle accommodating the diiron center, confirmed the four universally conserved glutamate residues (E) and the two universally conserved histidine (H) residues across plant and fungal sequences (S3 Fig). Amongst Angiosperms, only the AOX1d sequences from Monocots lacked the conserved CysII, having a serine residue instead. The conserved plant CysII was not found in any of the fungal sequences (S3 Fig).

### 3.3. *AOX* expression analysis in tomato root plants and *R*. *irregularis*

**Fig 4:**
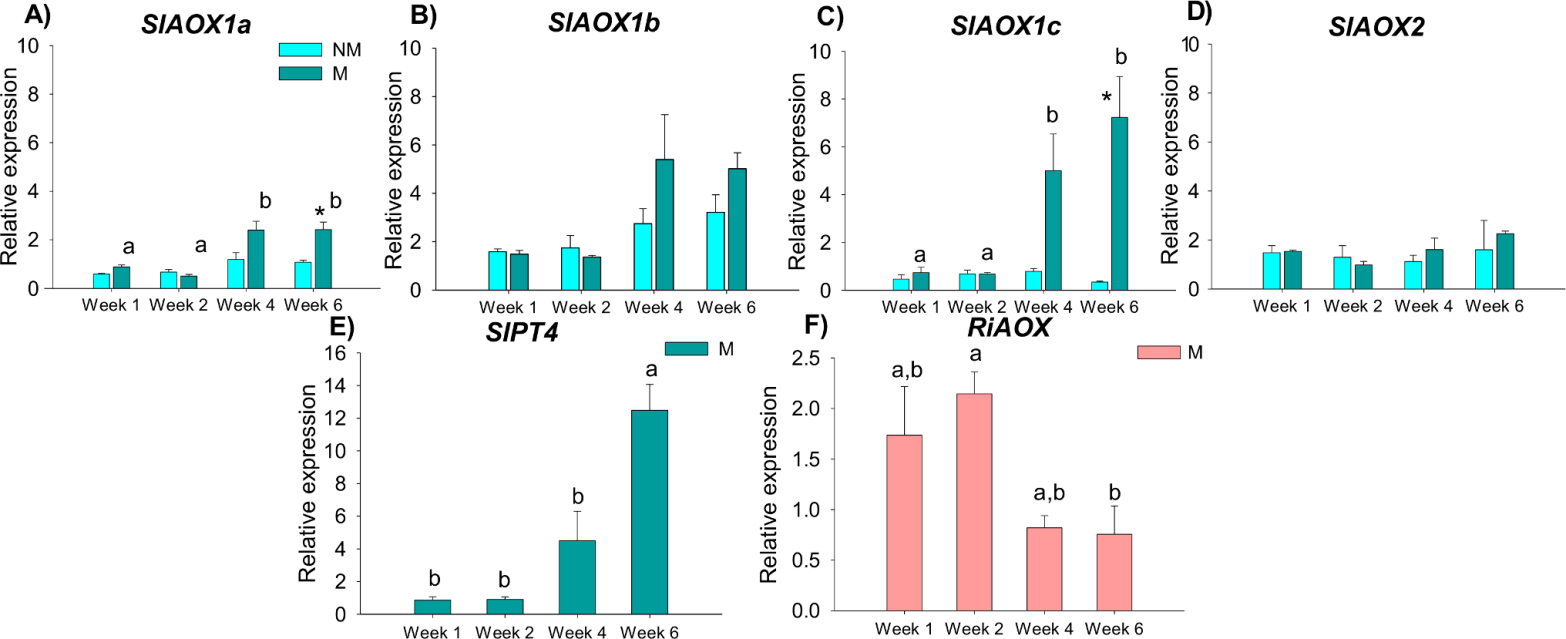
Expression levels of *AOX* gene family in mycorrhizal and non-mycorrhizal tomato roots. Expression levels were analysed at 1, 2, 4 and 6 weeks post-inoculation with *R*. *irregularis*. The expression levels of *SlAOX1a* (A), *SlAOX1b* (B), *SlAOX1c* (C), *SlAOX2* (D) and *SlPT4* (E) were analysed in roots of mycorrhizal and non-mycorrhizal tomato plants. The transcript levels of *RiAOX* were analysed on mycorrhizal roots of tomato (F). Asterisks (*) indicate significant differences (*P* < 0.05) in expression between mycorrhizal and non-mycorrhizal roots at one time point. Different superscript letters ^(a, b)^ indicate significant differences (*P* < 0.05) between weeks on mycorrhizal roots. No expression of *SlPT4* and *RiAOX* was found in non-mycorrhizal roots.

The gene expression analysis revealed that tomato *AOX* genes were stable during the first two weeks of the experiment, although mycorrhizal colonisation was already visible one week post-inoculation (S2 Table). At four weeks post-inoculation, two genes were up-regulated by mycorrhization with *R*. *irregularis* (Fig 4): *SlAOX1a* and *SlAOX1c* gene transcript levels increased (Fig 4A and 4C), coinciding with the appearing of the first arbuscules and vesicules (S2 Table). By 6 weeks post-inoculation, when more than a half of the roots were colonized by the AMF (S2 Table), *SlAOX1a* and *SlAOX1c* transcript accumulation was significantly higher in mycorrhizal plant roots (Fig 4A and 4C) (*P* < 0.05), and a similar trend was found for *SlAOX1b* despite the non-significant results (Fig 4B). Non-significant differences were observed for *SlAOX2* along the entire experiment (Fig 4C). The expression of *SlAOX* genes in non-mycorrhizal plant roots remained stable during the 6 weeks of the trial. The tomato phosphate transporter *SlPT4* was 14.5 fold up-regulated between 1 and 6 weeks post-inoculation with *R*. *irregularis* (*P* < 0.001) (Fig 4E).

The *AOX* transcript levels of *R*. *irregularis* were relatively stable during the first two weeks post-inoculation, but then decreased by 2.8 fold until 6 weeks (*P* < 0.05) (Fig 4F). No expression of *SlPT4* and *RiAOX* was found in non-mycorrhizal plant roots.

### 3.4. High-throughput sequencing of *RiAOX*

#### 3.4.1. Molecular identification *of R*. *irregularis* spores

The phylogenetic analysis of the cloned sequences placed all spores in the *R*. *irregularis* clade, together with sequences retrieved from databases and already recognised as belonging to *R*. *irregularis* (S4 Fig). The *R*. *intraradices* group was completely separated from *R*. *irregularis*.

#### 3.4.2. High throughput sequencing of *AOX* in *R*. *irregularis* single spores

A total of 385 379 high quality reads were obtained from the 9 individual spores, with a mean percentage of mapped reads to the reference sequence of 97%. The mean length of mapped read in the 9 spores was of 143 bp (see details on Table 1). The mean variant coverage was of 3 856 and the mean SNV coverage was of 3 482 (Table 1).

**Table 1:** Reads and variants statistics of the 9 *R*. *irregularis* single spores.

Sample	# of reads	% of mapped reads	Average mapped read length	Average variant coverage	Average SNV coverage
**INOQ 2**	71 940	98	138	5 598	4 714
**INOQ 3**	4 456	98	105	276	226
**INOQ 5**	112 690	98	142	9 308	8 707
**BEG144_2**	5 521	95	136	436	381
**BEG144_3**	49 936	97	156	4 847	4 297
**BEG144_8**	36 707	96	144	3 282	3 028
**BEG72_1**	41 944	98	155	4 509	3 810
**BEG72_2**	28 258	98	156	2 861	2 689
**BEG72_4**	33 927	98	152	3 585	3 485
**TOTAL**	385 379				
**Mean**	42 820	97	143	3 856	3 482

**Fig 5:**
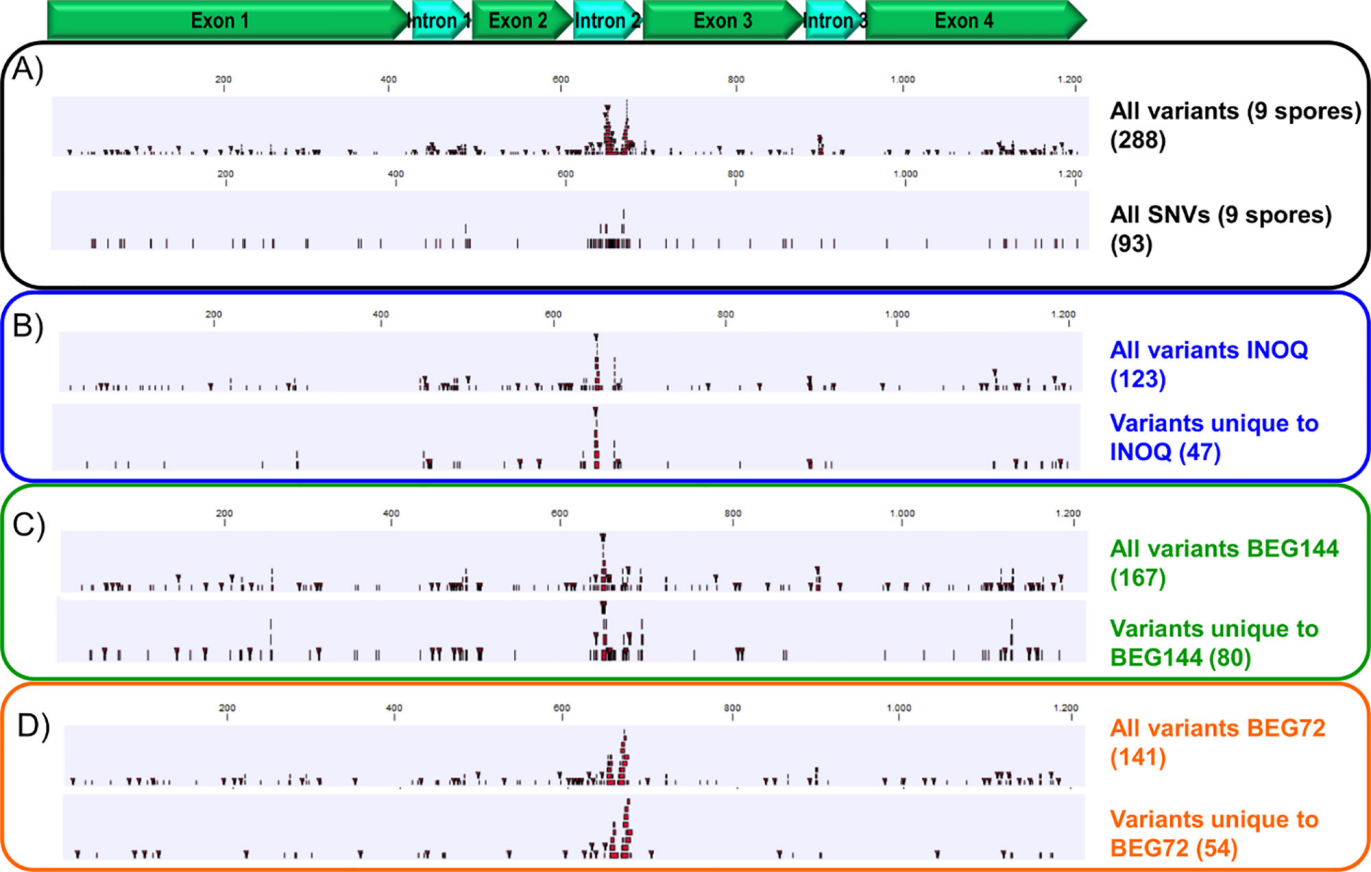
Distribution of variants along the 1209 bp *AOX* sequence *of R*. *irregularis*. All the 288 variants (SNVs, MNVs, deletions, insertions and replacements) and only the SNVs present in the 9 spores are shown in A). The variants present in INOQ, BEG144 and BEG72 isolates plus the variants unique to each isolate are presented in B), C) and D), respectively.

When considering all 9 spores, a total of 288 nucleotide variants (including SNVs, MNVs, insertions, deletions and replacements) were found within the 1209 bp sequence length (Fig 5A). Of these 288 variants, 93 corresponded to SNVs (Fig 5A). Many polymorphisms accumulated in the region corresponding to intron 2: 92 of which 38 were SNVs; however, a large number were also found dispersed throughout the sequence, including all exons (Fig 5A, S5 Fig). Only 13 polymorphisms, consisting mostly of insertions or deletions of 1 bp and one SNV were identical between spores.

In the three spores of the INOQ isolate, a total of 123 variants were identified, of which 47 were unique to this isolate (16% of the 288 variants) (Fig 5B). Regarding the BEG144 isolate, a total of 167 variants were found and 80 were exclusive to this isolate (28% of the 288 variants) (Fig 5C). For BEG72, 141 polymorphisms were found and 54 (19% of the 288 variants) were unique to this isolate (Fig 5D). When comparing the three spores from the same isolate, the percentage of unique polymorphisms in each spore varied with isolate: 33% (13% corresponding to SNVs), 37% (17% for SNVs) and 26% (7% for SNVs) for INOQ, BEG144 and BEG72, respectively.

**Fig 6:**
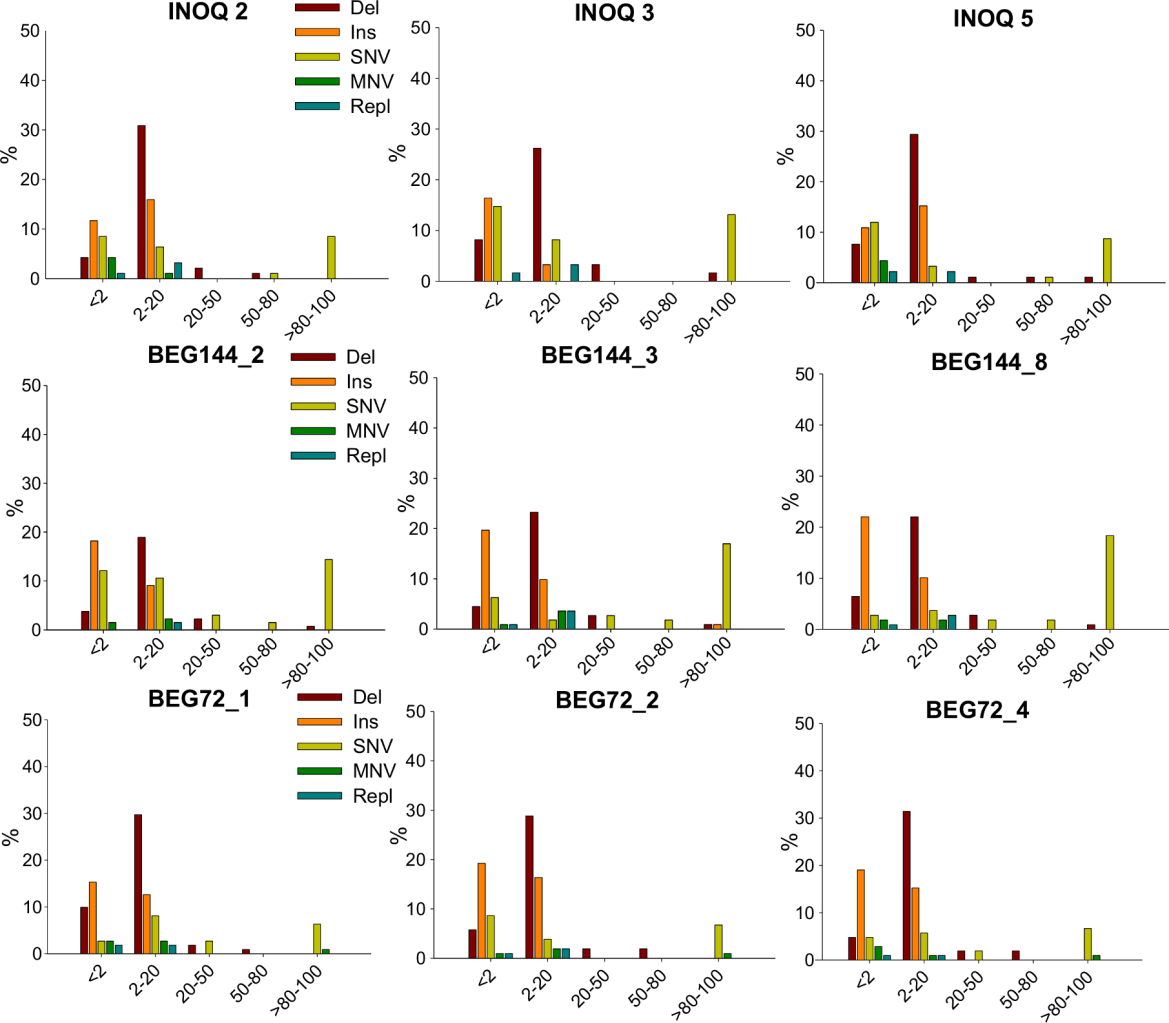
Variants frequency (deletions, insertions, SNVs, MNVs and replacements) distribution on single spores of INOQ, BEG144 and BEG72 isolates. Variants can range from less than 2% until almost 100% of frequency of occurrence (relatively to the total number of variants in one spore).

**Fig 7:**
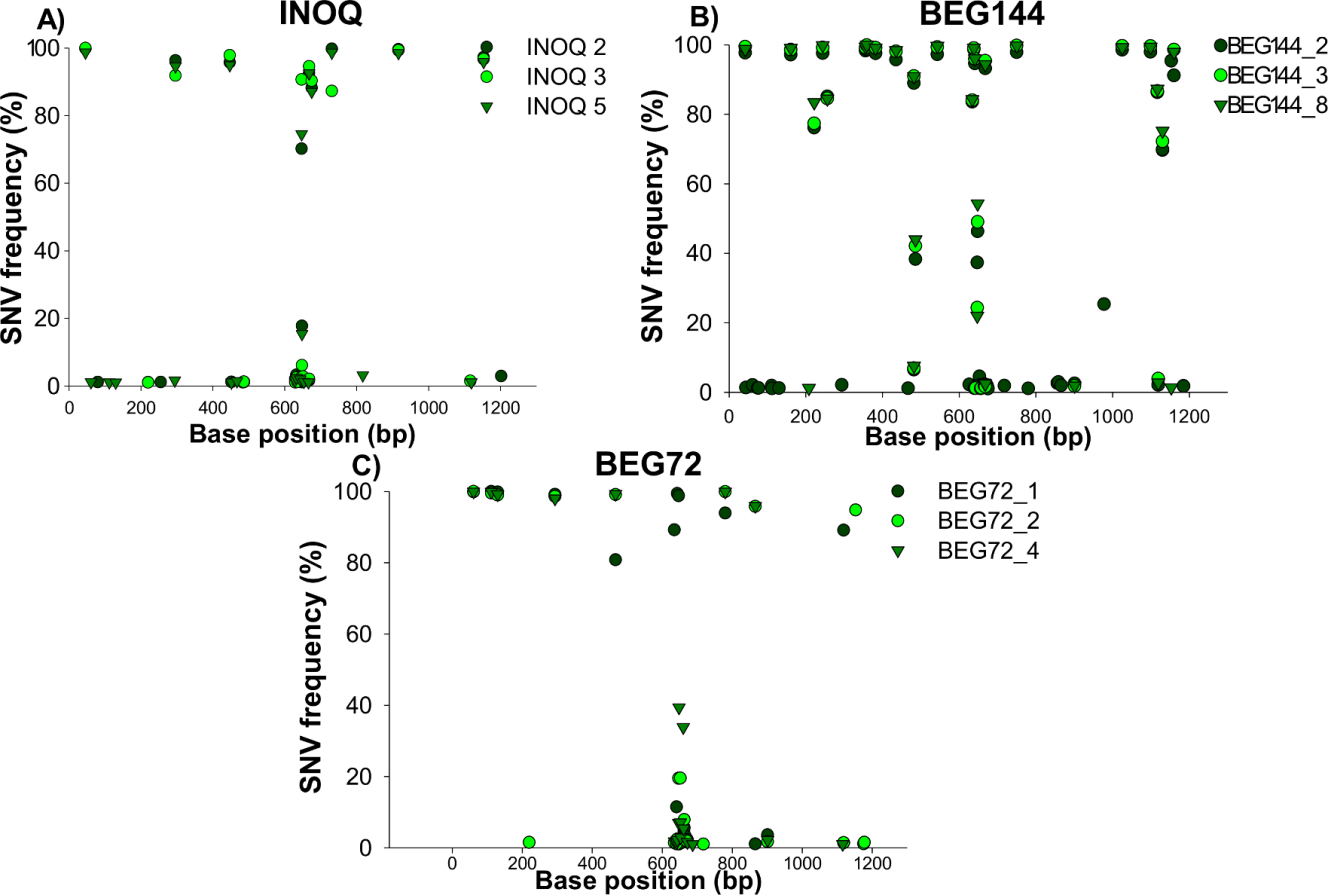
Frequency of SNVs along the *RiAOX* sequence on the INOQ, BEG144 and BEG72 isolates.

The frequency (read count divided by read coverage) distribution of all variants (in relation to the total number of variants in one spore) was plotted on histograms, ranging from rare (<2% of frequency of occurrence) until variants that showed a frequency of occurrence of almost 100% (Fig 6). All isolates presented highly frequent polymorphisms (>80–100% of frequency of occurrence), particularly SNVs (Fig 6). Nevertheless, most of variants showed until 20% of frequency of occurrence. The frequency of SNVs was relatively similar amongst isolates, ranging from low frequency SNVs until highly frequent SNVs, with several of them showing frequencies close to 100% (Fig 7A–7C).

**Fig 8:**
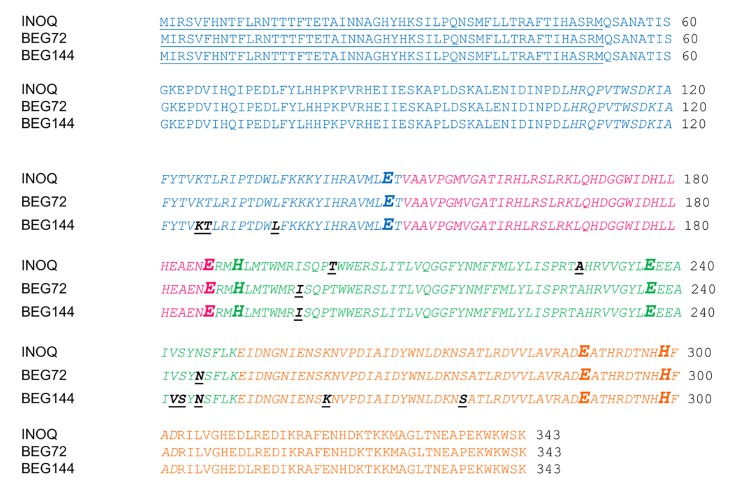
Location of the putative amino acid changes induced by SNVs or MNVs within the AOX ferritin-like domain, in the INOQ, BEG72 and BEG144 isolates. Exons are indicated by different colours. The 52 bp of the predicted N-terminal are underlined. The AOX ferritin-like domain, obtained by nucleotide search on CDD database is italicised. The putative amino acid changes are bold and underlined. The location of the four universally conserved glutamate residues (E) and the two universally conserved histidine residues (H) are presented in bold and enlarged.

Variants (SNVs and MNVs) that could induce putative aa substitutions were compared amongst spores (Fig 8, S3 Table, S6 Fig). These SNVs and MNVs ranged from low until highly frequent (S3 Table). Most of other type of variants induced frameshift mutations and not aa changes. In INOQ 2, 7 SNVs and one MNV that could lead to aa changes were identified, being located in exons 1, 3 and 4. One SNV was located in the AOX ferritin-like domain on exon 3. Five SNVs were identified in INOQ 3 spore (one within the AOX ferritin-like domain), and 10 variants (8 SNVs and 2 MNVs) in the INOQ 5 (two SNVs within the AOX ferritin-like domain) (Fig 8). BEG144 isolate showed 24, 12 and 13 SNVs or MNVs in spores 2, 3 and 8, respectively. Of these, several were located in the same positions of exons 1, 3 and 4 (Fig 8) and 9 were within the AOX domain. BEG72_1 showed 8 variants that could induce aa changes, and two were located in exon 3, inside the AOX ferritin-like domain. BEG72_2 and BEG72_4 showed 10 and 7 variants, respectively, of which two (either SNV or MNV) were located in the same position as in BEG72_1, in the exon 3 (Fig 8).

## Discussion

### 4.1. Characterisation of *RiAOX* gene and corresponding translated peptide

The comparison of *R*. *irregularis AOX* genomic sequence with other fungi revealed a high variability in the gene structure, not only related with the number of exons/introns but also with their sizes (Fig 1). This differs greatly with respect to plant *AOX* genes, with a well-known structure typically composed by four exons with well conserved sizes. Only exceptionally, *AOX* plant genes present variation on exons number, and consequently on their size, which is due to the occurrence of events related with intron loss or gain [38,39]. The cause and the effects of the variation on number and length of fungal *AOX* introns that we observed in the present study is unknown; but in fact, fungi have been shown to possess extensive and rapid intron gains and losses, even in closely related species [40]. Data obtained from *Cryptococcus* and *Fusarium* genomes showed very recent intron gains and losses, indicating that internal intron gain and loss might be a general trend affecting fungal intron evolution over short evolutionary timeframes [41]. Moreover, a high number of variable intron positions were also found in several genes of the *Cryptococcus* and *Fusarium* clades [41].

*RiAOX* has four exons and three introns (Fig 1). This agrees with the mean number of 2.46 introns in *R*. *irregularis* genome [42]. However, for instance the microsporidian *S*. *lophii* does not have any intron on the *AOX* gene. Microsporidians seem to have almost [43] or completely [44] lost introns in their genomes, concomitantly with a low efficiency of splicing of the remaining ones. In the genome of *S*. *lophii* only 8 putative intron-containing genes were found [45], and *AOX* is not among them. In contrast, *AOX* gene of *A*. *bisporus* showed 10 introns. *A*. *bisporus* has been described as the preferred model for adaptation, persistence, and growth in a humic-rich environment, and has particular genetic and enzymatic mechanisms governing adaptation to the niche formed during plant degradation [46]. Since introns enable alternative splicing and often encode small RNAs such as miRNAs [47], thus allowing a finer level of regulatory control, differences in intron number might be related with individual lifestyle and particular adaptations of fungal species. New introns may be fixed by selection if part of the coding sequence has negative effects on the function of the protein (e.g., deleterious mutation). In this case, splicing of the affected sequence may remedy the deleterious effects [48]. Also, the presence of introns may provide benefits under stress conditions and play an important role in adapting growth to various conditions [49]. Whether this is an explanation for the high number of introns in *A*. *bisporus AOX* we cannot say; however it might be that variations in fungal *AOX* gene intron number relate not only with genetic similarity but also with habitat and its constraints.

The predicted amino acid sequence of the *RiAOX* gene revealed one of the largest N-termini analysed in the present study, comparable to that of some Ascomycota. This region, located N-terminally to the first hydrophobic domain, has long been recognised as variable compared to the rest of the protein amongst different organisms [50,51]. There is also a length difference between prokaryotic and eukaryotic AOX sequences since N-termini of eukaryotic AOX are much longer than their prokaryotic counterparts [51,52]. In the present study, only eukaryotic AOX proteins were analysed, and the predicted N-termini that can support mitochondrial targeting sequence and the cleavage site showed a great size range amongst fungal species (Fig 2). Interestingly, from the three Microsporidia species analysed, only one displayed a predictable AOX N-terminal (*T*. *hominis*), which was also the smallest N-terminal found along with that of *R*. *allomycis* (Fig 2). Microsporidians lack canonical mitochondria, having mitosomes instead [53], and also lack Krebs cycle and a respiratory chain, but retain an alternative respiratory pathway which however seems to be differentially regulated along their life cycle [54]. These facts might be related with the non-existence or the short size of the predicted AOX N-terminal that we found in microsporidians. However, and similarly to bacterial AOX [52], the essential information in AOX of microsporidians, *R*. *allomycis* and some Zygomycota might be contained in a minimal N-terminal or then is unnecessary for the cell-membrane targeting.

#### 4.1.1. Regulatory motifs in fungi AOX

Multiple alignments of AOX sequences revealed that N-termini of fungal and plant proteins are highly divergent, particularly on helix *α*1 and on the region containing the plant conserved CysI (S1 Fig). In plants, CysI and CysII are involved in the mechanism of *α*-keto acid (particularly pyruvate) regulation [55,56]. The more N-terminal CysI acts as a site for intermolecular bond formation and *α*-keto acid activation [57], but CysII has a less well defined role in this activation. None of these cysteines were found in fungi, indicating a different mode of AOX regulation. Also animals lack the N-terminal cysteine, important for plant AOX regulation [11]. Crichton et al. [25] showed also that the E/DNV motif may be crucial during pyruvate activation in non-thermogenic plants. It was suggested that the presence or not of a conserved CysI residue and/or E/DNV motif, and a preferential stimulation by pyruvate or succinate may be related to the thermogenic (or not) phenotype of plants [58]. Interestingly however, our results showed that *R*. *allomycis* has the ENV motif, identical to most of the plant species, and RiAOX presents the ENS motif, identical to some eudicot AOX1d. Contrarily to plants, AOXs of fungi are generally monomeric and subjected to post-translational regulation by purine nucleoside 5’ monophosphates GMP and/or AMP, and its induction is much dependent on cytochrome pathway restriction and triggering by ROS [51]. Therefore, the reasons for this similarity with plants remain elusive, but it does not seem likely that it indicates any similarity in regulation with plants.

#### 4.1.2. Phylogenetic analysis of *R*. *irregularis* AOX

The results obtained in the present study showed a clear division between plant and fungal AOX protein sequences (Fig 3). In plants, AOX is encoded by a small gene family composed by a maximum of six gene members distributed in two subfamilies, the AOX1 and AOX2-subfamily (Cardoso et al. 2015). Our results are in accordance with the recent plant AOX classification scheme proposed by Costa et al. [37]. AOX sequences were completely separated between the two AOX subfamilies, the AOX2 (only described in eudicot plant species) and AOX1-subfamily, and amongst the AOX1 it created three main groups (AOX1a-c/1e, AOX1d monocots and AOX1d eudicots).

Concerning fungi AOX, our results clearly show the existence of two major clades: the Dikarya subkingdom, which includes Basidiomycota and Ascomycota phyla and a clade comprising all the other studied phyla (Fig 3). The fungal AOX phylogenetic tree therefore resembles the general phylogeny constructed for fungi [59], thus seeming that AOX phylogeny echoes fungi lineage-specific events.

#### 4.1.3. Phylogeny of AOX in fungi

The position of *R*. *irregularis* AOX and of its sister group formed by members of the Zygomycota (sub-phylum Mucoromycotina, order Mucorales) (Fig 3) reflects the phylogenetic relation described by Ebersberger et al. [59], Tisserant et al. [42] and Lin et al. [60], where the Glomeromycota and particularly *R*. *irregularis* are referred as close to Mucorales. Interestingly, Tisserant and colleagues [42] have compared the genome of *R*. *irregularis* with Mucoromycotina and Chytridiomycota genomes and found similar patterns of absence of several genes related to diverse metabolisms and detoxification/stress responses, suggesting that this loss is a common feature of the early diverging fungi. However, whereas for instance Lin and colleagues define Mucoromycotina as the closest fungi to *R*. *irregularis*, when regarding only AOX sequences *R*. *irregularis* seems more related to Chytridiomycota and Cryptomycota. *R*. *irregularis* is an obligate biotrophic symbiont and *R*. *allomycis* is an endobiotic biotrophic parasite; whether the relative proximity between AOX of *R*. *irregularis* and *R*. *allomycis* indicates some resemblance in lifestyle is unknown but can be hypothesised. In any case, *R*. *irregularis* AOX sequence seems to relate with that of ancient fungi, given the fact that *R*. *allomycis* has been placed on the putative primary branch of the fungi, basal to Chytridiomycota [61].

Although the division between the clade containing *R*. *irregularis* and the sister clade of Zygomycota was clear, their position relatively to Microsporidia was unresolved. Nevertheless, a relatively close AOX phylogenetic relationship between ancient fungi and microsporidians seems to be supported by findings of James et al. [62], who related *R*. *allomycis* with Microsporidia through shared signatures of parasitism and genomic features.

### 4.2. Putative role of *AOX* genes in AM symbiosis

AOX has already been described as participating in the regulation of growth, development and resistance against oxidative stress in the Ascomycota plant pathogen *Sclerotinia sclerotiorum* [19] and in the transition from mycelium to the yeast form in the human pathogen *Paracoccidioides brasiliensis* [18]. Our results from the tomato mycorrhization trial also point to a dynamic involvement of *RiAOX* in a crucial part of *R*. *irregularis* lifecycle–plant root mycorrhization. This relates to the reported by Besserer and co-workers [22], who showed that AMF AOX respiratory chain is involved in early steps of symbiosis, mainly during spore germination and hyphal branching of *Gigaspora rosea*. These pre-contact phases are followed by the infection of roots via fungal appressoria, the subsequent colonisation of the cortex by fungal hyphae, and finally the formation of arbuscules. The further progress of the symbiosis is characterised by permanent arbuscule build-up and break-down [2,3]. In our study, by 6 weeks post-inoculation the number of arbuscules was high and transcript levels of *RiAOX* had decreased (Fig 4F), suggesting that *RiAOX* is implicated in the early phases of the symbiotic process.

In tomato, *AOX* family is composed by four gene members, three *AOX1* (*SlAOX1a*, *SlAOX1b*, *SlAOX1c*) and one *AOX2* subfamily members [63]. Similarly to other plants, also in tomato different reports show the involvement of *SlAOX1* in the response to biotic and abiotic stress conditions [63,64]. *AOX2*, only found in eudicot plant species has been described as typically constitutive or related to development (see review in Cardoso et al. [65]). In the present work, a constitutive expression was found for all the four *SlAOX* genes analyzed, though at lower levels in the non-mycorrhizal plant roots, (Fig 4A–4D). *SlAOX1a* and *SlAOX1c* expression (and at a lower extent *SlAOX1b*) in mycorrhizal plants seemed to correlate with the development of *R*. *irregularis* arbuscules within the root, as transcript levels increased after 4 and especially after 6 weeks of inoculation, whereas *SlAOX2* showed a similar pattern between mycorrhizal and non-mycorrhizal plants. The pattern of transcript accumulation observed in mycorrhizal plant roots for both *SlAOX1a and SlAOX1c* genes, which seems to occur simultaneously with the arbuscules establishment, is coincident with the pattern observed for the AM-specific tomato phosphate transporter *SlPT4*, which also correlated with mycorrhizal development. Phosphate (Pi) plays a central role in virtually all major metabolic processes in higher plants and algae, including photosynthesis and respiration [66,67] and enhanced uptake of inorganic Pi is one of the major benefits that plants derive from AMF. Therefore, the increase in the transcript accumulation of *SlPT4* here identified would be related with the development of mycorrhiza and the synthesis of phosphate transport proteins by the plant to uptake the Pi supplied by the AMF. Yip and Vanlerberghe [68] investigated the existence of a potential involvement of AOX during resupply of P to P-limited cells, which is accompanied by a dramatic stimulation of respiratory O_2_ consumption. They measured O_2_ consumption rates upon the use of cytochrome or alternative pathway inhibitors in wild type and transgenic cell lines lacking AOX, and provided an *in vivo* evidence that AOX has a role during the period of high respiration supporting the Pi uptake, which seems to dampen the mitochondrial generation of active oxygen species. Therefore, the increase in both *SlAOX1a* and *SlAOX1c* observed in the present work can be hypothesized as linked to an increase in cell respiration in the arbusculated cells to support the continuous increment in Pi released into the cell as a consequence of root colonisation, while the AMF *AOX* would be mainly associated to the early stages of the plant-fungus interaction.

### 4.3. High throughput sequencing of *RiAOX* reveals intra and inter-isolate spore variability

AMF do not pass through a uninucleate stage in any part of their life cycle and mature AMF spores contain hundreds of nuclei obtained directly from the parent mycelium (e.g. [7]). In the AMF *Glomus etunicatum* a very high level of allelic variation per single spore was found for a low copy number gene [69]. Interestingly, these authors found that an individual spore always contains fewer alleles than the overall allelic diversity observed when eight spores were pooled together. This is in agreement with the results obtained in the present work. When considering all the 9 spores from the three independent isolates, we found a total of 288 *AOX* nucleotide variants (Fig 5), but the maximum number of variants found within one spore was of 132, for BEG144_2. Moreover, the total number of variants found for the three spores of BEG144 was of 167, which is superior to the number of variants of the spore BEG144_2. Similar results were found for the other isolates. Such differences in AMF genetic variation among sister spores may arise from a genetic bottleneck occurring during sporulation [69]. Indeed, segregation of genetically differentiated nuclei can lead to different genomic contents between subcultures from the same isolate. *R*. *irregularis* isolate (DAOM197198) has been maintained in several different *in vitro* culture collections since 1992 [70]. Cardenas-Flores et al. [71] found that subcultures from the same isolate which had been maintained in separate laboratories for 69 generations anastomosed (or exchanged nuclei through hyphal fusion) at a much lower rate than spores taken from within the same isolate and subculture. This could indicate that subcultures no longer share the same genomic content at a given gene, since decreased rates of anastomosis were observed between genetically diverse isolates [8,72].

In the present study, the three *R*. *irregularis* isolates showed different patterns and numbers of exclusive *AOX* polymorphisms’ for each spore. The BEG72 isolate showed a lower percentage (26%) of unique variants for each of the spores than INOQ (33%) or BEG144 (37%). The three isolates have distinct geographic origins and BEG144 was originally isolated in 1979, BEG72 in 1996 [32] and INOQ in 1999 [73]. The higher or lower homogeneity within isolates is likely to be highly related with its origin and environmental constraints, particularly important due to AOX relatedness with biotic and abiotic stress responses. Also, when comparing the number of variants from the three isolates, it is not possible to establish any relation with time since initial isolation or *in vitro* propagation, as the number of variants from the initial population is unknown. Without replenishment of genetic variation during repeated propagation, segregation may lead to a loss of nuclear variants with each new generation. The only means of countering this loss is through the action of anastomosis, in which hyphal strands fuse and nuclei are exchanged [74]. Interestingly, it was shown that genetic exchange in AMF can also be beneficial for plant growth, and that symbiosis-specific gene transcription is altered by such genetic exchange [75].

A recent study from Lin and co-workers [60] brought new insights to the question of how genetic diversity is organized within *R*. *irregularis* spores; interestingly, they have found low levels of polymorphisms genome wide between nuclei of *R*. *irregularis*, but in contrast, within a single nucleus, the 45S rDNA repeat unit was found to be quite divergent. They found evidence of a basically homokaryotic genome of the *R*. *irregularis* studied strain, though it might happen that slightly divergent nuclei can still coexist [60]. The gene copy number of *AOX* in *R*. *irregularis* genome is unknown, so we cannot say whether the variants found in the present study come from single or multiple nuclei, or from a combination of both. Nevertheless, given the fact that the number of polymorphisms found when considering the three spores from each isolate was higher than in individual spores, it seems that there is diversity loss in individual spores. Also in our study, due to the amplicon target approach, the average variant coverage was much higher than the average sequencing depth reported by these authors so likely we were prone to find more polymorphisms.

In spite of the high polymorphic diversity that was found in the spores, it seems that a high number of *AOX* variants are under functional constraints, since many occurred in intron 2 or induced frameshift mutations. However, since some putative amino acid changes induced by SNVs or MNVs were found within the AOX conserved domain (Fig 8), it might happen that some of these variants may be subjected to positive selective pressure within the population. AMF show sufficient phenotypic variation and fitness differentials for selection to occur [76]. It has been proposed that direct selection on fungal traits related to their survival and performance in the soil independent of the host is likely the major driver of differentiation for AMF, with evidences for direct fungal responses to soil conditions such as pH, hypoxia, and temperature [76]. However, the precise origins of selective pressures on *RiAOX* sequences and possible consequences of sequence variability for protein function remain to be determined.

## Considerations

The drive of this research was the confirmation of assumptions required for functional marker development: gene involvement and ample sequence variability. First, our results suggest that *R*. *irregularis AOX* is involved in the establishment of the symbiosis with plant roots. At plant level, *AOX* appears more involved on the later stages, when arbuscules are already established and the Pi uptake inside the cell is high. Second, we found relatively high levels of allelic diversity within isolates that could further be tested as functional or related with phenotypic differences. Indeed it seems that *AOX* genetic diversity exists not only inter but also intra-spore: thus different spores do not necessarily contain the same genetic variation. Also, given *AOX* relatedness with biotic and abiotic stress responses, the differences in gene variants amongst different *R*. *irregularis* isolates are likely to be related with its origin and environmental constraints. Considering these findings, and from an applied perspective, the next steps would be (1) functionally testing the variants and try to establish a link between genotype and phenotype (for a potential technological approach, see [77,78] and (2) analyse the scope for selection at spore level for inoculum production towards a high quality and homogeneous inoculum.

## Supporting Information

S1 Fig Multiple alignments of the hydrophobic region of AOX in plants and fungi (helices *α*1 and *α*4). The conserved regulatory cysteine residue (CysI) is located N-terminal to *α*1. Some sequences lack the conserved CysI, having a serine residue instead. The conserved CysI is not found in any of the fungal sequences (boxed). The fungal and plant sequences are fairly divergent N-terminal, whereas the helix *α*4 is more conserved. *R*. *irregularis* AOX is dashed.
(TIF)

S2 Fig Multiple alignments on region 3 of AOX in fungi and plants. Plants generally present the glutamic acid/aspartic acid, asparagine and valine (E/DNV) motif (with exceptions of AOX1d from monocots and some AOX1d from eudicots). The fungal sequences are much more divergent. RiAOX showed the ENS motif, identical to that of some AOX1d from eudicots.
(TIF)

S3 Fig Conserved Fe-Fe ligands in the AOX helices *α*2, *α*3, *α*5 and *α*6. The four universally conserved glutamate residues and the two universally conserved histidine residues are indicated by asterisks (*). The conserved cysteine residue seen in Angiosperms (CysII) is located on helix *α*2. Amongst Angiosperms, only the AOX1d sequences from Monocots lack the conserved CysII, having a serine residue instead. The conserved CysII is not found in any of the fungal sequences (boxed).
(TIF)

S4 Fig Phylogenetic analysis (NJ) of clones from ribosomal rRNA sequences of the ITS-LSU-SSU region from the 9 *R*. *irregularis* single spores. All spores placed in the *R*. *irregularis* clade, together with sequences retrieved from databases and already recognized as belonging to *R*. *irregularis*. The *R*. *intraradices* group was completely separated from *R*. *irregularis*.
(PDF)

S5 Fig Location of all variants (SNVs, MNVs, insertions, deletions, replacements) of *RiAOX* in the INOQ, BEG144 and BEG72 spores. (TIF)

S6 Fig Location of the variants that putatively can induce changes on *R*. *irregularis* AOX protein sequence, in the INOQ, BEG144 and BEG72 isolates. The location of the SNVs is indicated with red lines.
(TIF)

S1 List Accession numbers of fungal and plant AOX sequences used for phylogenetic analysis. (PDF)

S1 Table *S*pecific primers used for *qPCR* in tomato (Sl) and *R*. *irregularis* (Ri). Sequences accession numbers and amplicon sizes are presented.
(PDF)

S2 Table Mycorrhizal colonisation of tomato roots. Tomato roots were inoculated with *R*. *irregularis* over a period of six weeks and evaluated microscopically with trypan-blue. Scores according to the method of Trouvelot *et al*. (1986) are given for frequency (F %), intensity (M %) and relative intensity (m %) of mycorrhizal colonisation. Scores for the total number of vesicles (V %) and arbuscules (A %) are also given. Data represent the means of 4 replicates (± SE). Data not sharing a common superscript letter differ significantly (*P* < 0.05).
(PDF)

S3 Table Variants (SNVs and MNVs) that could induce putative amino acid substitutions in the 9 single spores. The variants within the AOX ferritin-like domain are in red.
(PDF)

## References

[1] Smith JE. Mycorrhizal Symbiosis. (Third Edition). Soil Sci Soc Am J 2009;73:694 10.2136/sssaj2008.0015br

[2] Parniske M. Arbuscular mycorrhiza: the mother of plant root endosymbioses. Nat Rev Microbiol 2008;6:763–75. 10.1038/nrmicro1987 18794914

[3] Harrison MJ. Signaling in the arbuscular mycorrhizal symbiosis. Annu Rev Microbiol 2005;59:19–42. 10.1146/annurev.micro.58.030603.123749 16153162

[4] Garg N, Chandel S. Arbuscular mycorrhizal networks: process and functions. A review. 2010;30:581–99.

[5] Baar J. From Production to Application of Arbuscular Mycorrhizal Fungi in Agricultural Systems: Requirements and Needs In: Varma A., editor. Mycorrhiza, Springer-Verlag Berlin Heidelberg; 2008, p. 361–73.

[6] Hoeksema JD, Chaudhary VB, Gehring C a., Johnson NC, Karst J, Koide RT, et al A meta-analysis of context-dependency in plant response to inoculation with mycorrhizal fungi. Ecol Lett 2010;13:394–407. 10.1111/j.1461-0248.2009.01430.x 20100237

[7] Koch AM, Croll D, Sanders IR. Genetic variability in a population of arbuscular mycorrhizal fungi causes variation in plant growth. Ecol Lett 2006;9:103–10. 10.1111/j.1461-0248.2005.00853.x 16958874

[8] Marleau J, Dalpé Y, St-Arnaud M, Hijri M. Spore development and nuclear inheritance in arbuscular mycorrhizal fungi. BMC Evol Biol 2011;11:51 10.1186/1471-2148-11-51 21349193 PMC3060866

[9] Kuhn G, Hijri M, Sanders IR. Evidence for the evolution of multiple genomes in arbuscular mycorrhizal fungi. Nature 2001;414:745–8. 10.1038/414745a 11742398

[10] Vicente C, Schneider C, Tavares de Sousa M, Arnholdt-Schmitt B. Can *AOX* be applied as functional marker in AMF communities? First Int. AOX Symp., 2008, p. 58.

[11] McDonald AE, Vanlerberghe GC, Staples JF. Alternative oxidase in animals: unique characteristics and taxonomic distribution. J Exp Biol 2009;212:2627–34. 10.1242/jeb.032151 19648408

[12] Joseph-Horne T, Hollomon DW, Wood PM. Fungal respiration: A fusion of standard and alternative components. Biochim Biophys Acta—Bioenerg 2001;1504:179–95. 10.1016/S0005-2728(00)00251-6 11245784

[13] Magnani T, Soriani FM, Martins VP, Nascimento AM, Tudella VG, Curti C, et al Cloning and functional expression of the mitochondrial alternative oxidase of *Aspergillus fumigatus* and its induction by oxidative stress. FEMS Microbiol Lett 2007;271:230–8. 10.1111/j.1574-6968.2007.00716.x 17425662

[14] Vanlerberghe GC. Alternative oxidase: A mitochondrial respiratory pathway to maintain metabolic and signaling homeostasis during abiotic and biotic stress in plants. Int J Mol Sci 2013;14:6805–47. 10.3390/ijms14046805 23531539 PMC3645666

[15] Arnholdt-Schmitt B, Costa JH, de Melo DF. AOX—a functional marker for efficient cell reprogramming under stress? Trends Plant Sci 2006;11:281–7. 10.1016/j.tplants.2006.05.001 16713324

[16] Cardoso HG, Arnholdt-Schmitt B. Functional Marker Development Across Species in Selected Traits In: Lübberstedt T T & Varshney RK, editor. Diagnostics Plant Breed., Springer Netherlands; 2013, p. 467–515. 10.1007/978-94-007-5687-8_21

[17] Akhter S, Mcdade HC, Gorlach JM, Heinrich G, Cox GM, Perfect JR. Role of alternative oxidase gene in pathogenesis of *Cryptococcus neoformans* 2003;71:5794–802. 10.1128/IAI.71.10.5794 PMC201089 14500501

[18] Martins VP, Dinamarco TM, Soriani FM, Tudella VG, Oliveira SC, Goldman GH, et al Involvement of an alternative oxidase in oxidative stress and mycelium-to-yeast differentiation in *Paracoccidioides brasiliensis* . Eukaryot Cell 2011;10:237–48. 10.1128/EC.00194-10 21183691 PMC3067407

[19] Xu T, Yao F, Liang W-S, Li Y-H, Li D-R, Wang H, et al Involvement of alternative oxidase in the regulation of growth, development, and resistance to oxidative stress of *Sclerotinia sclerotiorum* . J Microbiol 2012;50:594–602. 10.1007/s12275-012-2015-7 22923107

[20] Thomazella DPT, Teixeira PJPL, Oliveira HC, Saviani EE, Rincones J, Toni IM, et al The hemibiotrophic cacao pathogen *Moniliophthora perniciosa* depends on a mitochondrial alternative oxidase for biotrophic development. New Phytol 2012;194:1025–34. 10.1111/j.1469-8137.2012.04119.x 22443281 PMC3415677

[21] Tamasloukht MB, Se N, Kluever A, Jauneau A, Roux C. Root factors induce mitochondrial-related gene expression and fungal respiration during the developmental switch from asymbiosis to presymbiosis in the arbuscular mycorrhizal fungus *Gigaspora rosea* 2003;131:1468–78. 10.1104/pp.012898.ment PMC166906 12644696

[22] Besserer A, Bécard G, Roux C, Séjalon-Delmas N. Role of mitochondria in the response of arbuscular mycorrhizal fungi to strigolactones. 2009;4:75–7. 10.1104/pp.108.121400 PMC2634080 19704715

[23] Karlin S, Altschul SF. Applications and statistics for multiple high-scoring segments in molecular sequences. Proc Natl Acad Sci U S A 1993;90:5873–7. 10.1073/pnas.90.12.5873 8390686 PMC46825

[24] Tamura K, Stecher G, Peterson D, Filipski A, Kumar S. MEGA6: Molecular evolutionary genetics analysis version 6.0. Mol Biol Evol 2013;30:2725–9. 10.1093/molbev/mst197 24132122 PMC3840312

[25] Crichton PG, Affourtit C, Albury MS, Carré JE, Moore AL. Constitutive activity of *Sauromatum guttatum* alternative oxidase in *Schizosaccharomyces pombe* implicates residues in addition to conserved cysteines in α-keto acid activation. FEBS Lett 2005;579:331–6. 10.1016/j.febslet.2004.10.107 15642340

[26] Hewitt EJ. Sand and water culture methods used in the study of plant nutrition. Tech. Commun. no. 22. Commonw. Agric. Bur., London: 1966.

[27] Phillips JM, Hayman DS. Improved procedures for clearing roots and staining parasitic and vesicular-arbuscular mycorrhizal fungi for rapid assessment of infection. Trans Br Mycol Soc 1970;55:158–IN18. 10.1016/S0007-1536(70)80110-3

[28] Trouvelot A, Kough JL G-P V. Mesure du taux de mycorhization VA d’un système radiculaire. Recherche de méthodes d’estimation ayant une signification fonctionnelle In: Gianinazzi-Pearson V. and Gianinazzi S., editor. Physiol. Genet. Asp. Mycorrhizae., Paris: INRA Press; 1986, p. 217–21.

[29] Martín Rodriguez JÁ, León Morcillo R, Vierheilig H, Antonio Ocampo J, Ludwig-Müller J, García Garrido JM. Mycorrhization of the *notabilis* and *sitiens* tomato mutants in relation to abscisic acid and ethylene contents. J Plant Physiol 2010;167:606–13. 10.1016/j.jplph.2009.11.014 20079554

[30] Vandesompele J, De Preter K, Pattyn F, Poppe B, Van Roy N, De Paepe A, et al Accurate normalization of real-time quantitative RT-PCR data by geometric averaging of multiple internal control genes. Genome Biol 2002;3:RESEARCH0034 10.1186/gb-2002-3-7-research0034 12184808 PMC126239

[31] Radonić A, Radonić A, Thulke S, Thulke S, Mackay IM, Mackay IM, et al Guideline to reference gene selection for quantitative real-time PCR. Biochem Biophys Res Commun 2004;313:856–62. 10.1016/j.bbrc.2003.11.177 14706621

[32] Camprubí A, Calvet C. Isolation and screening of mycorrhizal fungi from citrus nurseries and orchards and inoculation studies. HortScience 1996;31:366–9.

[33] Krüger M, Stockinger H, Krüger C, Schüssler A. DNA-based species level detection of *Glomeromycota*: one PCR primer set for all arbuscular mycorrhizal fungi. New Phytol 2009;183:212–23. 10.1111/j.1469-8137.2009.02835.x 19368665

[34] Stockinger H, Walker C, Schüßler A. “*Glomus intraradices* DAOM197198”, a model fungus in arbuscular mycorrhiza research, is not *Glomus intraradices* . New Phytol 2009;183:1176–87. 10.1111/j.1469-8137.2009.02874.x 19496945

[35] Stockinger H, Krüger M, Schüssler A. DNA barcoding of arbuscular mycorrhizal fungi. New Phytol 2010;187:461–74. 10.1111/j.1469-8137.2010.03262.x 20456046

[36] Saitou N, Nei M. The neighbor-joining method: a new method for reconstructing phylogenetic trees. Mol Biol Evol 1987;4:406–25. citeulike-article-id:93683. 3447015 10.1093/oxfordjournals.molbev.a040454

[37] Costa JH, McDonald AE, Arnholdt-Schmitt B, Fernandes de Melo D. A classification scheme for alternative oxidases reveals the taxonomic distribution and evolutionary history of the enzyme in angiosperms. Mitochondrion 2014;19:172–83. 10.1016/j.mito.2014.04.007 24751423

[38] Polidoros AN, Mylona PV, Arnholdt-Schmitt B. *AOX* gene structure, transcript variation and expression in plants. Physiol Plant 2009;137:342–53. 10.1111/j.1399-3054.2009.01284.x 19781002

[39] Considine MJ, Holtzapffel RC, Day D a, Whelan J, Millar a H. Molecular distinction between alternative oxidase from monocots and dicots. Plant Physiol 2002;129:949–53. 10.1104/pp.004150 12114550 PMC1540239

[40] Torriani SFF, Stukenbrock EH, Brunner PC, McDonald BA, Croll D. Evidence for extensive recent intron transposition in closely related fungi. Curr Biol 2011;21:2017–22. 10.1016/j.cub.2011.10.041 22100062

[41] Croll D, McDonald BA. Intron gains and losses in the evolution of *Fusarium* and *Cryptococcus* fungi. Genome Biol Evol 2012;4:1148–61. 10.1093/gbe/evs091 23054310 PMC3514964

[42] Tisserant E, Malbreil M, Kuo A, Kohler A, Symeonidi A, Balestrini R, et al Genome of an arbuscular mycorrhizal fungus provides insight into the oldest plant symbiosis. Proc Natl Acad Sci U S A 2013;110:20117–22. 10.1073/pnas.1313452110 24277808 PMC3864322

[43] Keeling PJ, Slamovits CH. Simplicity and complexity of microsporidian genomes. Eukaryot Cell 2004;3:1363–9. 10.1128/EC.3.6.1363-1369.2004 15590811 PMC539024

[44] Cuomo CA, Desjardins CA, Bakowski M., Goldberg J, Ma AT, Becnel JJ, et al. Microsporidian genome analysis reveals evolutionary strategies for obligate intracellular growth. 2012:2478–88. 10.1101/gr.142802.112 PMC3514677 22813931

[45] Campbell SE, Williams TA, Yousuf A, Soanes DM, Paszkiewicz KH, Williams BAP. The genome of *Spraguea lophii* and the basis of Host-Microsporidian interactions. PLoS Genet 2013;9:1–15. 10.1371/journal.pgen.1003676 PMC3749934 23990793

[46] Morin E, Kohler A, Baker AR. Correction for Morin et al., Genome sequence of the button mushroom *Agaricus bisporus* reveals mechanisms governing adaptation to a humic-rich ecological niche. Proc Natl Acad Sci U S A 2013;110:4146 10.1073/pnas.1300201110 PMC3491501 23045686

[47] Bartel DP. MicroRNAs: Genomics, Biogenesis, Mechanism, and Function. Cell 2004;116:281–97. 10.1016/S0092-8674(04)00045-5 14744438

[48] Irimia M, Rukov JL, Penny D, Vinther J, Garcia-Fernandez J, Roy SW. Origin of introns by “intronization” of exonic sequences. Trends Genet 2008;24:378–81. 10.1016/j.tig.2008.05.007 18597887

[49] Parenteau J, Durand M, Véronneau S, Lacombe A-A, Morin G, Cecez B, et al. Deletion of many yeast introns reveals a minority of genes that require splicing for function. 2008;19:1932–41. 10.1091/mbc.E07 PMC2366882 18287520

[50] Umbach AL, Siedow JN. The cyanide-resistant alternative oxidases from the fungi *Pichia stipitis* and *Neurospora crassa* are monomeric and lack regulatory features of the plant enzyme. Arch Biochem Biophys 2000;378:234–45. 10.1006/abbi.2000.1834 10860541

[51] McDonald AE. Alternative oxidase: An inter-kingdom perspective on the function and regulation of this broadly distributed “cyanide-resistant” terminal oxidase. Funct Plant Biol 2008;35:535–52. 10.1071/FP08025 32688810

[52] Finnegan PM, Umbach AL, Wilce JA. Prokaryotic origins for the mitochondrial alternative oxidase and plastid terminal oxidase nuclear genes. FEBS Lett 2003;555:425–30. 10.1016/S0014-5793(03)01309-7 14675750

[53] Williams BAP, Hirt RP, Lucocq JM, Embley TM. A mitochondrial remnant in the microsporidian *Trachipleistophora hominis* . Nature 2002;418:865–9. 10.1038/nature00949 12192407

[54] Dolgikh VV, Senderskiy IV, Pavlova OA, Naumov AM, Beznoussenko GV. Immunolocalization of an alternative respiratory chain in *Antonospora* (*Paranosema*) *locustae* spores: Mitosomes retain their role in microsporidial energy metabolism. Eukaryot Cell 2011;10:588–93. 10.1128/EC.00283-10 21296913 PMC3127642

[55] Umbach AL, Gonzàlez-Meler MA, Sweet CR, Siedow JN. Activation of the plant mitochondrial alternative oxidase: Insights from site-directed mutagenesis. Biochim Biophys Acta—Bioenerg 2002;1554:118–28. 10.1016/S0005-2728(02)00219-0 12034477

[56] Umbach AL, Siedow JN. The reaction of the soybean cotyledon mitochondrial cyanide-resistant oxidase with sulfhydryl reagents suggests that α-keto acid activation involves the formation of a thiohemiacetal. J Biol Chem 1996;271:25019–26. 10.1074/jbc.271.40.25019 8798784

[57] Rhoads DM, Umbach AL, Sweet CR, Lennon AM, Rauch GS, Siedow JN, et al Regulation of the cyanide-resistant alternative oxidase of plant mitochondria. J Biol Chem 1998;273:30750–6. 9804851 10.1074/jbc.273.46.30750

[58] Moore AL, Shiba T, Young L, Harada S, Kita K, Ito K. Unraveling the heater: new insights into the structure of the alternative oxidase. Annu Rev Plant Biol 2013;64:637–63. 10.1146/annurev-arplant-042811-105432 23638828

[59] Ebersberger I, De Matos Simoes R, Kupczok A, Gube M, Kothe E, Voigt K, et al A consistent phylogenetic backbone for the fungi. Mol Biol Evol 2012;29:1319–34. 10.1093/molbev/msr285 22114356 PMC3339314

[60] Lin K, Limpens E, Zhang Z, Ivanov S, Saunders DGO, Mu D, et al Single nucleus genome sequencing reveals high similarity among nuclei of an endomycorrhizal fungus. PLoS Genet 2014;10 10.1371/journal.pgen.1004078 PMC3886924 24415955

[61] James TY, Kauff F, Schoch CL, Matheny PB, Hofstetter V, Cox CJ, et al Reconstructing the early evolution of Fungi using a six-gene phylogeny. Nature 2006;443:818–22. 10.1038/nature05110 17051209

[62] James TY, Pelin A, Bonen L, Ahrendt S, Sain D, Corradi N, et al Shared Signatures of parasitism and phylogenomics unite cryptomycota and microsporidia. Curr Biol 2013;23:1548–53. 10.1016/j.cub.2013.06.057 23932404

[63] Holtzapffel RC, Castelli J, Finnegan PM, Millar AH, Whelan J, Day DA. A tomato alternative oxidase protein with altered regulatory properties. Biochim Biophys Acta—Bioenerg 2003;1606:153–62. 10.1016/S0005-2728(03)00112-9 14507436

[64] Fung RWM, Wang CY, Smith DL, Gross KC, Tao Y, Tian M. Characterization of alternative oxidase (*AOX*) gene expression in response to methyl salicylate and methyl jasmonate pre-treatment and low temperature in tomatoes. J Plant Physiol 2006;163:1049–60. 10.1016/j.jplph.2005.11.003 16376455

[65] Cardoso HG, Nogales A, Frederico AM, Svensson JT, Macedo ES, Valadas V, et al Natural *AOX* gene diversity In: Gupta KJ, Mur LAJ, Neelwarne B, editors. Altern. Respir. pathways High. plants., Oxford: John Wiley & Sons, Inc; 2015, p. 241–54.

[66] Plaxton WC, Tran HT. Metabolic adaptations of phosphate-starved plants. Plant Physiol 2011;156:1006–15. 10.1104/pp.111.175281 21562330 PMC3135920

[67] Theodorou ME, Elrifi IR, Turpin DH, Plaxton WC. Effects of phosphorus limitation on respiratory metabolism in the green alga *Selenastrum minutum* . Plant Physiol 1991;95:1089–95. 10.1104/pp.95.4.1089 16668095 PMC1077656

[68] Yip JYH, Vanlerberghe GC. Mitochondrial alternative oxidase acts to dampen the generation of active oxygen species during a period of rapid respiration induced to support a high rate of nutrient uptake. Physiol Plant 2001;112:327–33. 10.1034/j.1399-3054.2001.1120305.x 11473689

[69] Boon E, Zimmerman E, St-Arnaud M, Hijri M. Allelic differences within and among sister spores of the arbuscular mycorrhizal fungus *Glomus etunicatum* suggest segregation at sporulation. PLoS One 2013;8:e83301 10.1371/journal.pone.0083301 24386173 PMC3873462

[70] Chabot S, Bécard G, Piché Y. Life cycle of *Glomus intraradix* in root organ culture. Mycologia 1992;84:315–21. 10.2307/3760183

[71] Cárdenas-Flores A, Draye X, Bivort C, Cranenbrouck S, Declerck S. Impact of multispores in vitro subcultivation of *Glomus* sp. MUCL 43194 (DAOM 197198) on vegetative compatibility and genetic diversity detected by AFLP. Mycorrhiza 2010;20:415–25. 10.1007/s00572-009-0295-5 20082102

[72] Croll D, Giovannetti M, Koch AM, Sbrana C, Ehinger M, Lammers PJ, et al Nonself vegetative fusion and genetic exchange in the arbuscular mycorrhizal fungus *Glomus intraradices* . New Phytol 2009;181:924–37. 10.1111/j.1469-8137.2008.02726.x 19140939

[73] Hildebrandt U, Kaldorf M, Bothe H. The zinc violet and its colonization by arbuscular mycorrhizal fungi. J Plant Physiol 1999;154:709–17. 10.1016/S0176-1617(99)80249-1

[74] Bever JD, Wang M. Arbuscular mycorrhizal fungi: hyphal fusion and multigenomic structure. Nature 2005;433:E3–4; discussion E4. 10.1038/nature03295 15650700

[75] Colard A, Angelard C, Sanders IR. Genetic exchange in an arbuscular mycorrhizal fungus results in increased rice growth and altered mycorrhiza-specific gene transcription. Appl Environ Microbiol 2011;77:6510–5. 10.1128/AEM.05696-11 21784911 PMC3187136

[76] Helgason T, Fitter AH. Natural selection and the evolutionary ecology of the arbuscular mycorrhizal fungi (Phylum Glomeromycota). J Exp Bot 2009;60:2465–80. 10.1093/jxb/erp144 19429838

[77] Arnholdt-Schmitt B, Hansen LD, Nogales A. Calorespirometry, oxygen isotope analysis and functional-marker-assisted selection (‘CalOxy-FMAS') for genotype screening: A novel concept and tool kit for predicting stable plant growth performance and functional marker identification. Brief Funct Genomics 2015:1–6. 10.1093/bfgp/elv008 25818699

[78] Arnholdt-Schmitt B, Valadas V, Döring M. Functional marker development is challenged by the ubiquity of endophytes—a practical perspective. Brief Funct Genomics 2014:1–6. 10.1093/bfgp/elu049 25526729

