# Supplementary figures and images for: Intra and Inter-Spore Variability in Rhizophagus irregularis AOX Gene
Source: PLoS One. 2015 Nov 5;10(11):e0142339. doi: 10.1371/journal.pone.0142339 (PMC4634980; doi:10.1371/journal.pone.0142339)

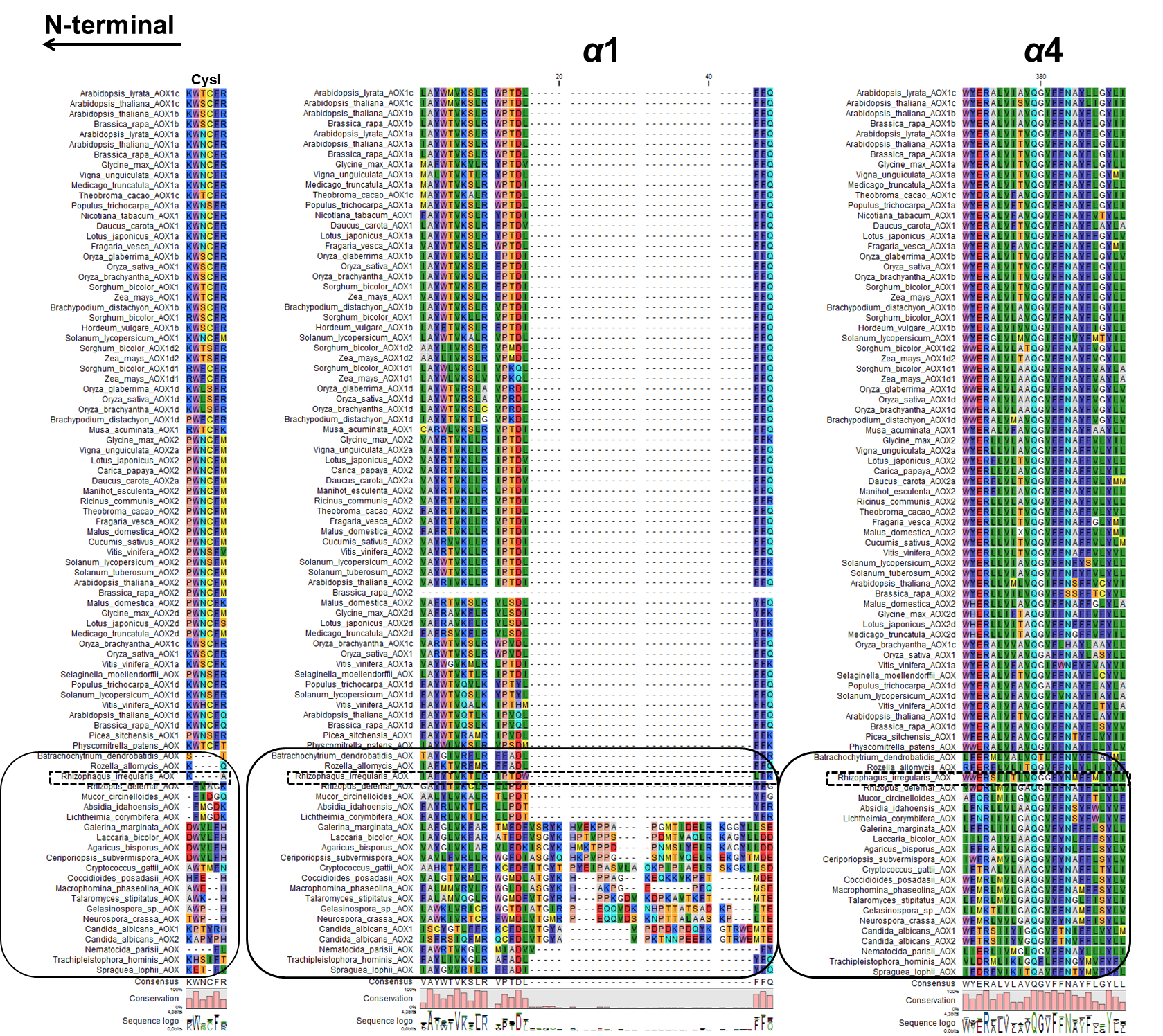

Supplement: S1 Fig — The conserved regulatory cysteine residue (CysI) is located N-terminal to α1. Some sequences lack the conserved CysI, having a serine residue instead. The conserved CysI is not found in any of the fungal sequences (boxed). The fungal and plant sequences are fairly divergent N-terminal, whereas the helix α4 is more conserved. R. irregularis AOX is dashed. (TIF) [file pone.0142339.s001.tif]

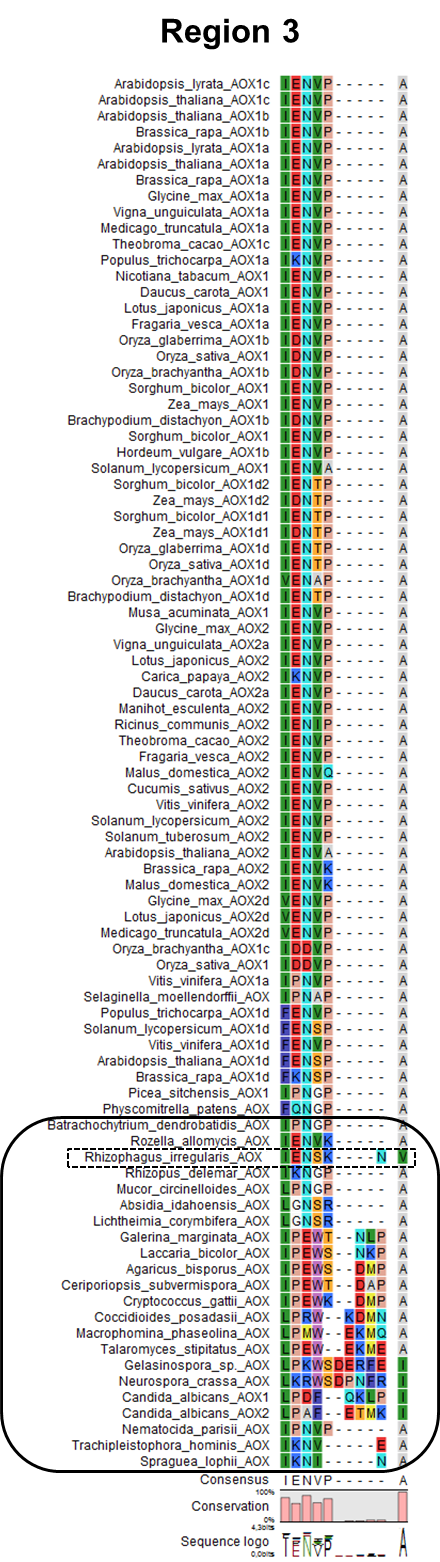

Supplement: S2 Fig — Plants generally present the glutamic acid/aspartic acid, asparagine and valine (E/DNV) motif (with exceptions of AOX1d from monocots and some AOX1d from eudicots). The fungal sequences are much more divergent. RiAOX showed the ENS motif, identical to that of some AOX1d from eudicots. (TIF) [file pone.0142339.s002.tif]

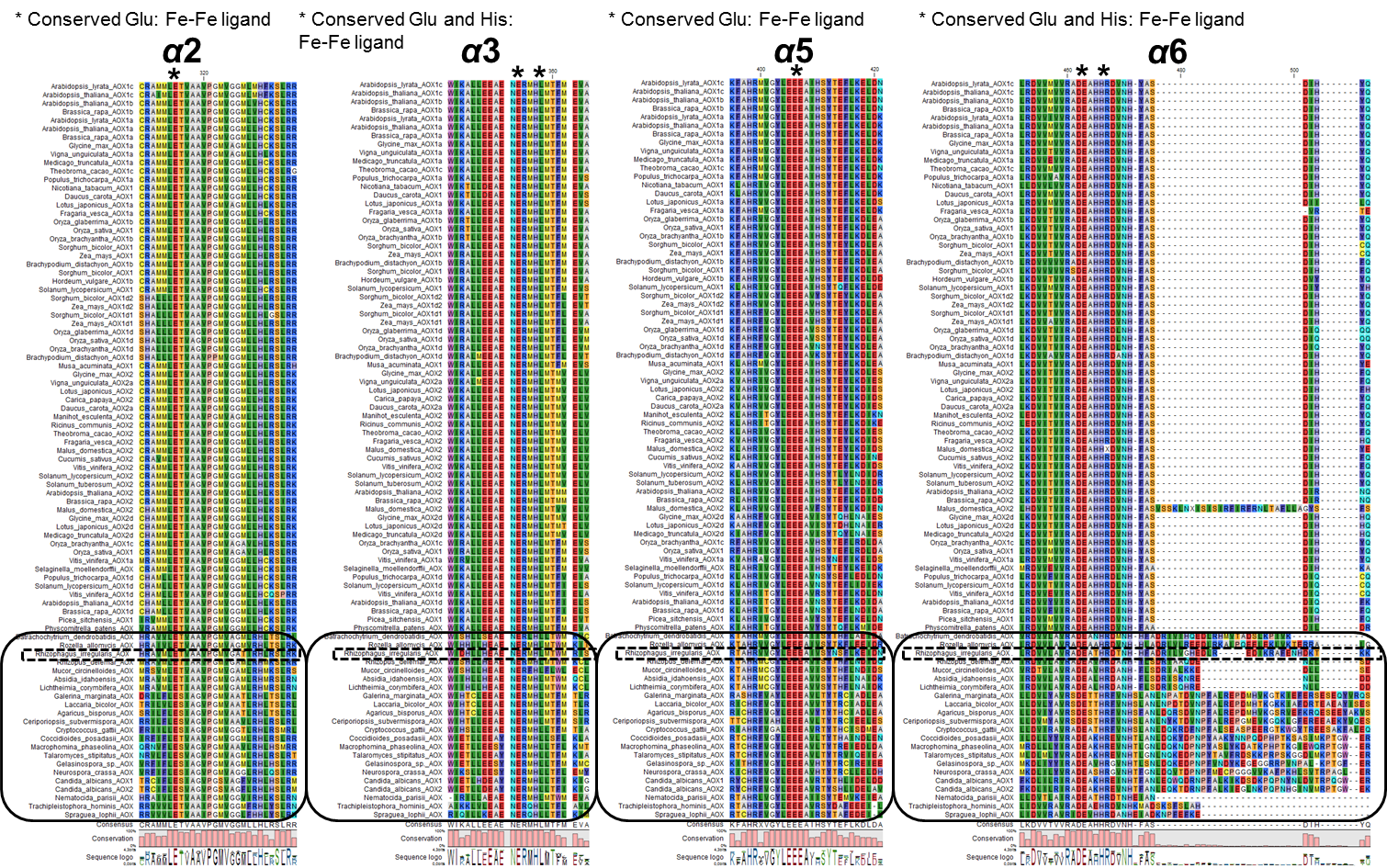

Supplement: S3 Fig — The four universally conserved glutamate residues and the two universally conserved histidine residues are indicated by asterisks (*). The conserved cysteine residue seen in Angiosperms (CysII) is located on helix α2. Amongst Angiosperms, only the AOX1d sequences from Monocots lack the conserved CysII, having a serine residue instead. The conserved CysII is not found in any of the fungal sequences (boxed). (TIF) [file pone.0142339.s003.tif]

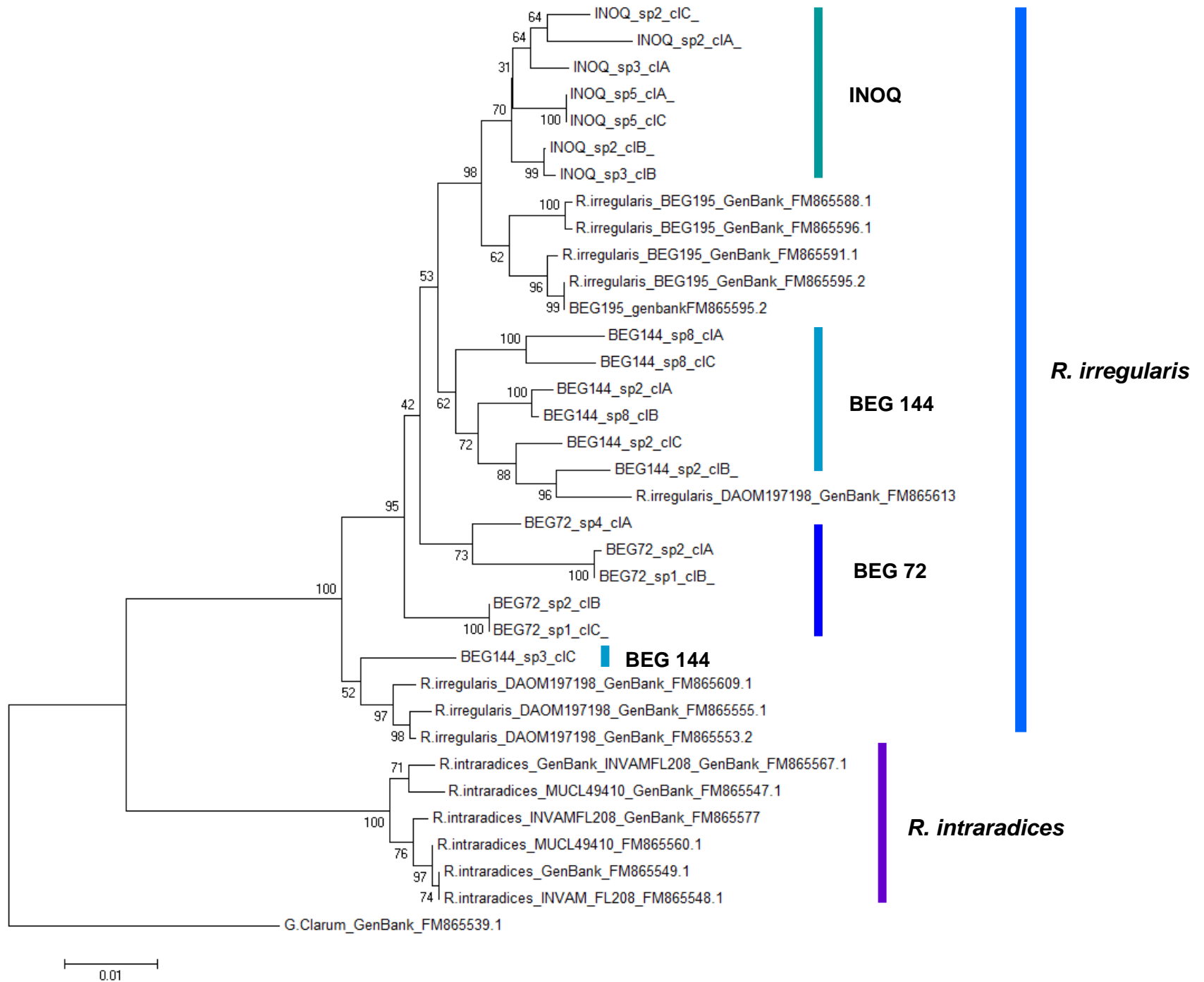

Supplement: S4 Fig — All spores placed in the R. irregularis clade, together with sequences retrieved from databases and already recognized as belonging to R. irregularis. The R. intraradices group was completely separated from R. irregularis. (PDF) [file pone.0142339.s004.pdf]

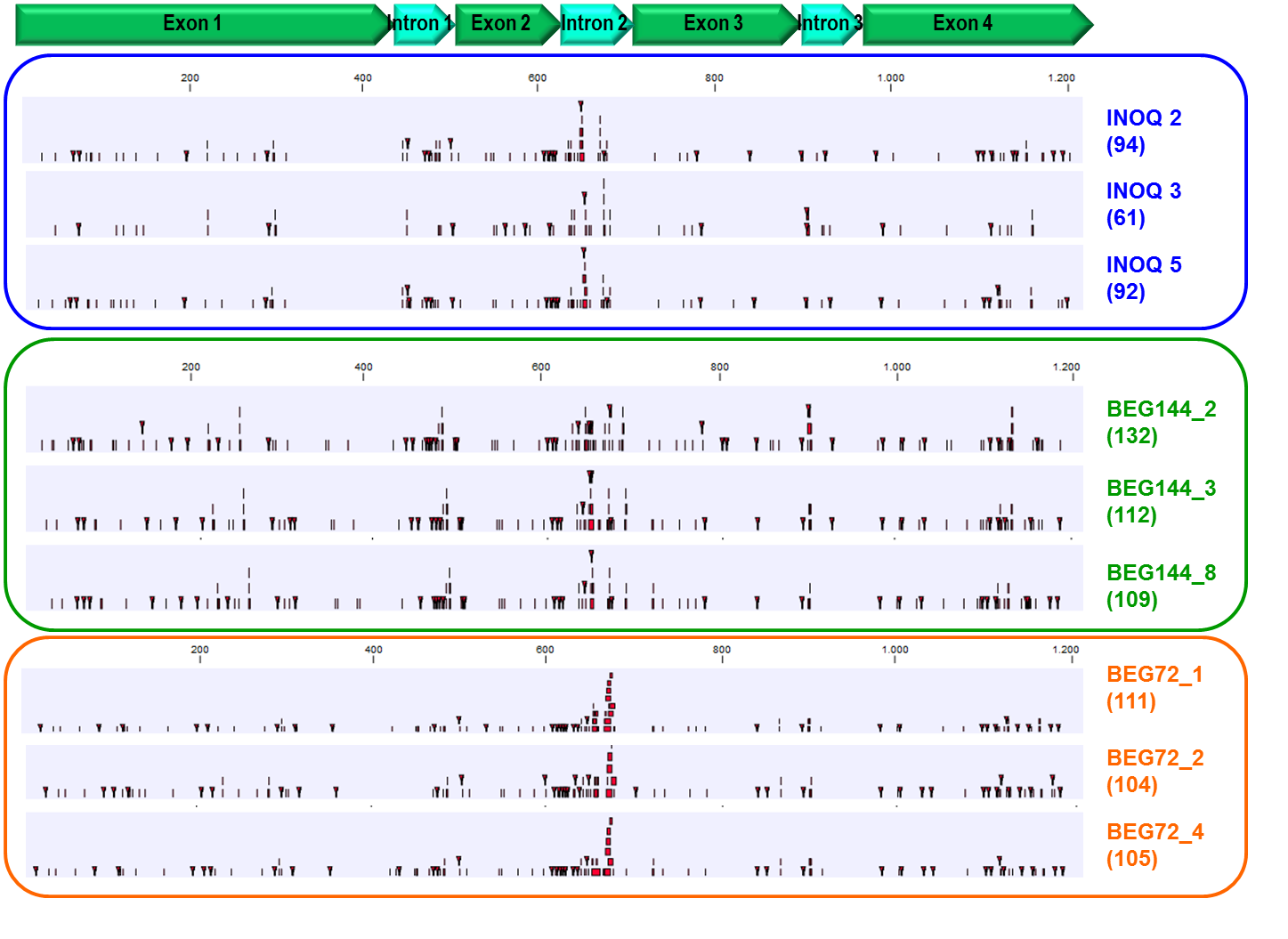

Supplement: S5 Fig — (TIF) [file pone.0142339.s005.tif]

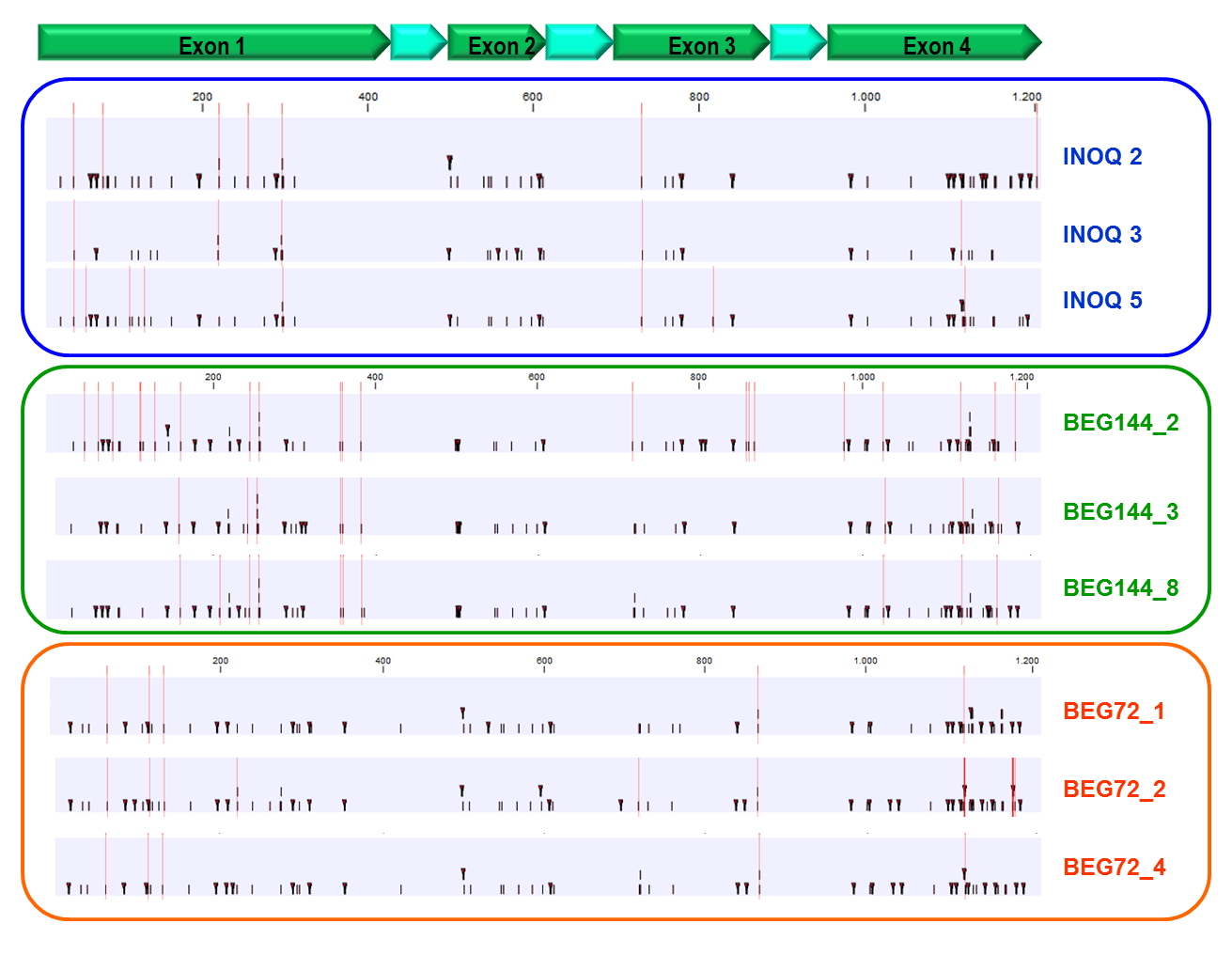

Supplement: S6 Fig — The location of the SNVs is indicated with red lines. (TIF) [file pone.0142339.s006.tif]
