# Supplementary material for: Intra and Inter-Spore Variability in Rhizophagus irregularis AOX Gene
Source: PLoS One. 2015 Nov 5;10(11):e0142339. doi: 10.1371/journal.pone.0142339 (PMC4634980; doi:10.1371/journal.pone.0142339)
Supplement: S1 List — (PDF) [file pone.0142339.s007.pdf]

FUNGI

>Galerina marginata_AOX_A0A067TS33

>Absidia_idahoensis_AOX_A0A077X462

>Rhizopus_delemar_AOX _1CQV1

>Ceriporiopsis_subvermispora_AOX _M2QSD3

>Laccaria_bicolor _B0CVV3

>Agaricus_bisporus_K5X8R3

>Cryptococcus_gattii_E6R040

>Talaromyces_stipitatus_B8LT09

>Coccidioides_posadasii_C5PHD7

>Macrophomina_phaseolina_K2S5D4

>Gelasinospora_sp_Q8J1Z2

>Neurospora_crassa_Q7S371

>Candida_albicans_AOX1_AAC98914.1

>Candida_albicans_AOX2_AAF21993.1

>Trachipleistophora_hominis_D5JAJ1

>Nematocida_parisii_I3EP00

>Spraguea_lophii_S7WC03

>Batrachochytrium_dendrobatidis_ F4P6T0

>Lichtheimia_corymbifera_CDH55114

>Mucor_circinelloides_S2JKA7

>Rozella_allomycis_CSF55

PLANTS

>Arabidopsis_lyrata_AOX1a_AL3G24690

>Arabidopsis_lyrata_AOX1c_AL5G06730

>Arabidopsis_thaliana_AOX1d_AT1G32350

>Arabidopsis_thaliana_AOX1a_AT3G22370

>Arabidopsis_thaliana_AOX1b_AT3G22360

>Arabidopsis_thaliana_AOX1c_AT3G27620

>Arabidopsis_thaliana_AOX2_AT5G64210

>Brachypodium_distachyon_AOX1b_BD3G52505

>Brachypodium_distachyon_AOX1d_BD5G20547

>Brassica_rapa_AOX1a_BR05G21590

>Brassica_rapa_AOX1b_BR01G28900

>Brassica_rapa_AOX1d_Bra010153

>Brassica_rapa_AOX2_BR09G06750

>Carica_papaya_AOX2_CP00042G00490

>Cucumis_sativus_AOX2_Q7Y1B3

>Daucus_carota_AOX1_AID15803.1

>Daucus_carota_AOX2a_ADB24724.1

>Fragaria_vesca_AOX2_FV5G21950

>Fragaria_vesca_AOX1a_FV5G29310

>Glycine_max_AOX1a_GM04G14800

>Glycine_max_AOX2d_GM08G07690

>Glycine_max_AOX2_GM08G07700

>Hordeum vulgare_AOX1b sG9CJ23

>Lotus_japonicus_AOX1a_LJ2G020780

>Lotus_japonicus_AOX2_LJ4G005280

>Lotus_japonicus_AOX2d_LJ4G005290

>Malus_domestica_AOX2_MD13G026910

>Malus_domestica_AOX2_MD00G081720

>Manihot_esculenta_AOX2_ME10292G00060

>Medicago_truncatula_AOX1a_MT5G026620

>Medicago_truncatula_AOX2d_MT5G070870

>Musa_acuminata_AOX1_MA06G01170

>Nicotiana_tabacum_AOX1_Q41224

>Oryza_brachyantha_AOX1b_OB04G30980

>Oryza_brachyantha_AOX1c_OB02G22630

>Oryza_brachyantha_AOX1d_OB04G30990

>Oryza_glaberrima_AOX1b_ORGLA04G0206000

>Oryza_glaberrima_AOX1d_ORGLA04G0206100

>Oryza_sativa_AOX1_BGIOSGA008063

>Oryza_sativa_AOX1_BGIOSGA014422

>Oryza_sativa_AOX1d_BGIOSGA014421

>Picea_sitchensis_AOX1_A9NRS5

>Populus_trichocarpa_AOX1d_PT03G10390

>Populus_trichocarpa_AOX1a_PT12G00160

>Physcomitrella_patens_AOX_PP00183G00030

>Ricinus_communis_AOX2_RC30063G00030

>Selaginella_moellendorffii_AOX_SM00020G03270

>Solanum_lycopersicum_AOX1_SL08G005550.1

>Solanum_lycopersicum_AOX1d_SL08G075550.1

>Solanum_lycopersicum_AOX2_Solyc01g105220.2

>Solanum_tuberosum_AOX2_ST01G042270

>Sorghum_bicolor_AOX1d1_SB06G027420

>Sorghum_bicolor_AOX1d2_SB06G027430

>Sorghum_bicolor_AOX1_SB04G030820

>Sorghum_bicolor_AOX1_SB06G027410

>Theobroma_cacao_AOX2_TC02G011670

>Theobroma_cacao_AOX1c_TC03G031300

>Vitis_vinifera_AOX2_VV00G00110

>Vigna_unguiculata_AOX1a_Q4F8G4

>Vigna_unguiculata_AOX2a_A2IBG3

>Vitis_vinifera_AOX1a_VV02G09050

>Vitis_vinifera_AOX1d_VV02G09030

>Zea_mays_AOX1_ZM02G05500

>Zea_mays_AOX1d1_ZM02G05490
>Zea_mays_AOX1d2_ZM02G05480

BACTERIA
>Rhodanobacter_sp._AOX_I4WCT1
>Afipia_felis_AOX_A0A090MMY7
