# Supplementary material for: Intra and Inter-Spore Variability in Rhizophagus irregularis AOX Gene
Source: PLoS One. 2015 Nov 5;10(11):e0142339. doi: 10.1371/journal.pone.0142339 (PMC4634980; doi:10.1371/journal.pone.0142339)
Supplement: S1 Table — Sequences accession numbers and amplicon sizes are presented. (PDF) [file pone.0142339.s008.pdf]

Gene name	GenBank accession #	Primer sequence (5’-3’)	Size (bp)
SlEF-1	X14449	SlEF-1-F: GATTGGTGGTATTGGAACTGTC<br>SlEF-1-R: AGCTTCGTGGTGCATCTC	133
SlActin2	EU408340	SlActin2-F: TTGCTGACCGTATGAGCAAG<br>SlActin2-R: GGACAATGGATGGACCAGAC	186
SlPT4	AY885651	SlLePT4-F: GAAGGGGAGCCATTTAATGTGG<br>SlLePT4-R: ATCGCGGCTTGTTTAGCATTTC	182
SlAOX1a	NP_001234117	SlAOX1aFw_701: TGGAAGTTGCAAAGCCAAATG<br>SlAOX1aRev_757:CGCCTTGCACTGCGAAA	57
SlAOX1b	NP_001234120	SlAOX1bFw_817: GCCGATGCAACGCTAAAAG<br>SlAOX1bRev_879:TCCCGATGATGTGCCTCAT	62
SlAOX1c	XP_004244430	SlAOX1cFw_900: TGAGGGAAATATTGAAAACGTAGCT<br>SlAOX1cRev_961: GCGTCAAACGCCAATAATCA	62
SlAOX2	XP_004230603	SlAOX2Fw_366: CATGCCATGGGAGACATATCAG<br>SlAOX2Rev_426: GGGCACATGGTGCTTGCT	61
RiAOX	KT423115	RiAOXFw1: AAAATGAACGTATGCACTTGATGAC<br>RiAOXRv1: GCGTTCCCACCAGGTAGGT	62
RiTEF1a	AJ831587.1	RiTEF1a_Fw: TGAAAAAGAAGCACAAGAAC<br>RiTEF1a_Rv:CCAACACCCAAGCATA	57
RiBTub1	AY326320.1	RiBTub1_Fw: AAGCGGAATCTTGTGATTGTTTG<br>RiBTub1_Rv: CCCATACCAGCTCCAGTACCA	76
