# Supplementary material for: Intra and Inter-Spore Variability in Rhizophagus irregularis AOX Gene
Source: PLoS One. 2015 Nov 5;10(11):e0142339. doi: 10.1371/journal.pone.0142339 (PMC4634980; doi:10.1371/journal.pone.0142339)
Supplement: S2 Table — Tomato roots were inoculated with R. irregularis over a period of six weeks and evaluated microscopically with trypan-blue. Scores according to the method of Trouvelot et al. (1986) are given for frequency (F %), intensity (M %) and relative intensity (m %) of mycorrhizal colonisation. Scores for the total number of vesicles (V %) and arbuscules (A %) are also given. Data represent the means of 4 replicates (± SE). Data not sharing a common superscript letter differ significantly (P < 0.05). (PDF) [file pone.0142339.s009.pdf]

	Weeks post inoculation			
	**1**	**2**	**4**	**6**
F (%)	8.3 ± 4.8[a]	18.9 ± 5.9[a]	48.3 ± 2.4[b]	51.1 ± 7.8[b]
M (%)	0.2 ± 0.1[a]	0.3 ± 0.1[a]	9.4 ± 3.8[b]	18.4 ± 5.8[c]
m (%)	0.9 ± 0.4[a]	1.6 ± 0.4[b]	19.1 ± 7.4[c]	34.4 ± 5.3[c]
V (%)	0.0 ± 0.0[a]	0.0 ± 0.0[a]	0.5 ± 0.4[b]	1.2 ± 0.6[c]
A (%)	0.0 ± 0.0[a]	0.0 ± 0.0[a]	0.8 ± 0.4[b]	1.7 ± 0.6[c]
