# Supplementary material for: Intra and Inter-Spore Variability in Rhizophagus irregularis AOX Gene
Source: PLoS One. 2015 Nov 5;10(11):e0142339. doi: 10.1371/journal.pone.0142339 (PMC4634980; doi:10.1371/journal.pone.0142339)
Supplement: S3 Table — The variants within the AOX ferritin-like domain are in red. (PDF) [file pone.0142339.s010.pdf]

	Region	Type	Reference	Allele	Count	Coverage	Frequency	Homopolymer	Amino acid change	Non-synonymous
INOQ 2	46	SNV	G	A	2685	2688	99,88839	No	CDS:p.Val16Ile	Yes
INOQ 2	80	SNV	T	C	31	2661	1,164976	No	CDS:p.Leu27Ser	Yes
INOQ 2	85..86	MNV	CA	AC	116	5371	2,159747	No	CDS:p.Gln29Thr	Yes
INOQ 2	220	SNV	C	T	62	5717	1,084485	No	CDS:p.Pro74Ser	Yes
INOQ 2	255	SNV	A	T	52	4638	1,121173	No	CDS:p.Lys85Asn	Yes
INOQ 2	296	SNV	G	A	7257	7533	96,33612	No	CDS:p.Ser99Asn	Yes
INOQ 2	731	SNV	A	C	7559	7575	99,78878	No	CDS:p.Asn194Thr	Yes
INOQ 2	1202	SNV	G	A	2	68	2,941176	No	CDS:p.Ala329Thr	Yes
INOQ 3	46	SNV	G	A	176	176	100	No	CDS:p.Val16Ile	Yes
INOQ 3	220	SNV	C	T	3	289	1,038062	No	CDS:p.Pro74Ser	Yes
INOQ 3	296	SNV	G	A	260	283	91,87279	No	CDS:p.Ser99Asn	Yes
INOQ 3	731	SNV	A	C	371	425	87,29412	No	CDS:p.Asn194Thr	Yes
INOQ 3	1116	SNV	T	G	3	200	1,5	No	CDS:p.Val300Gly	Yes
INOQ5	46	SNV	G	A	4042	4094	98,72985	No	CDS:p.Val16Ile	Yes
INOQ5	61	SNV	C	T	50	4338	1,152605	No	CDS:p.His21Tyr	Yes
INOQ5	85..86	MNV	CA	AC	149	7932	1,878467	No	CDS:p.Gln29Thr	Yes
INOQ5	112	SNV	G	A	107	9951	1,075269	No	CDS:p.Ala38Thr	Yes
INOQ5	130	SNV	T	C	110	10384	1,059322	No	CDS:p.Ser44Pro	Yes
INOQ5	296	SNV	G	A	11370	12016	94,62383	No	CDS:p.Ser99Asn	Yes
INOQ5	731	SNV	A	C	12717	12892	98,64257	No	CDS:p.Asn194Thr	Yes
INOQ5	817	SNV	G	A	488	15363	3,176463	No	CDS:p.Ala223Thr	Yes
INOQ5	1116..1117	MNV	TG	GT	83	7279	1,140267	No	CDS:p.Val300Gly	Yes
INOQ5	1119	SNV	G	T	41	3777	1,085518	No	CDS:p.Gly301Val	Yes

	Region	Type	Reference	Allele	Count	Coverage	Frequency	Homopolymer	Amino acid change	Non-synonymous
BEG144_2	44	SNV	C	T	4	291	1,37457	No	CDS:p.Ala15Val	Yes

BEG144_2	61	SNV	C	T	6	284	2,112676	No	CDS:p.His21Tyr	Yes
BEG144_2	77	SNV	T	A	5	403	1,240695	No	CDS:p.Ile26Asn	Yes
BEG144_2	85..86	MNV	CA	AC	11	569	1,933216	No	CDS:p.Gln29Thr	Yes
BEG144_2	111	SNV	A	T	12	634	1,892744	No	CDS:p.Arg37Ser	Yes
BEG144_2	112	SNV	G	A	7	619	1,130856	No	CDS:p.Ala38Thr	Yes
BEG144_2	130	SNV	T	C	8	653	1,225115	No	CDS:p.Ser44Pro	Yes
BEG144_2	161	SNV	G	A	549	565	97,16814	No	CDS:p.Ser54Asn	Yes
BEG144_2	244	SNV	A	C	531	544	97,61029	No	CDS:p.Ile82Leu	Yes
BEG144_2	256	SNV	G	A	441	518	85,13514	No	CDS:p.Ala86Thr	Yes
BEG144_2	256..257	MNV	GC	CA	9	518	1,737452	No	CDS:p.Ala86Gln	Yes
BEG144_2	356	SNV	A	G	578	588	98,29932	No	CDS:p.Lys119Arg	Yes
BEG144_2	359	SNV	C	T	573	581	98,62306	No	CDS:p.Thr120Ile	Yes
BEG144_2	382	SNV	C	T	478	490	97,55102	No	CDS:p.Leu128Phe	Yes
BEG144_2	718	SNV	A	T	8	417	1,918465	No	CDS:p.Ile190Phe	Yes
BEG144_2	856	SNV	G	C	14	534	2,621723	No	CDS:p.Val236Leu	Yes
BEG144_2	859	SNV	T	A	15	514	2,918288	No	CDS:p.Ser237Thr	Yes
BEG144_2	866	SNV	A	G	10	506	1,976285	No	CDS:p.Asn239Ser	Yes
BEG144_2	978	SNV	A	T	230	906	25,38631	No	CDS:p.Lys254Ile	Yes
BEG144_2	1025	SNV	T	A	961	975	98,5641	No	CDS:p.Ser270Thr	Yes
BEG144_2	1119	SNV	G	T	6	280	2,142857	No	CDS:p.Gly301Val	Yes
BEG144_2	1160	SNV	A	G	217	238	91,17647	No	CDS:p.Asn315Asp	Yes
BEG144_2	1163..1164	MNV	CA	AT	18	265	6,792453	No	CDS:p.His316Ile	Yes
BEG144_2	1185	SNV	C	T	2	112	1,785714	No	CDS:p.Ala323Val	Yes

BEG144_3	85..86	MNV	CA	AC	120	3493	3,435442	No	CDS:p.Gln29Thr	Yes
BEG144_3	161	SNV	G	A	4824	4892	98,60998	No	CDS:p.Ser54Asn	Yes
BEG144_3	244	SNV	A	C	6178	6228	99,19717	No	CDS:p.Ile82Leu	Yes
BEG144_3	256	SNV	G	A	4901	5798	84,52915	No	CDS:p.Ala86Thr	Yes
BEG144_3	256..257	MNV	GC	CA	98	5798	1,690238	No	CDS:p.Ala86Gln	Yes
BEG144_3	356	SNV	A	G	7146	7203	99,20866	No	CDS:p.Lys119Arg	Yes
BEG144_3	359	SNV	C	T	7047	7056	99,87245	No	CDS:p.Thr120Ile	Yes
BEG144_3	382	SNV	C	T	6189	6243	99,13503	No	CDS:p.Leu128Phe	Yes

BEG144_3	718..719	MNV	AT	TA	152	6135	2,477588	No	CDS:p.Ile190Tyr	Yes
BEG144_3	1025	SNV	T	A	6262	6283	99,66576	No	CDS:p.Ser270Thr	Yes
BEG144_3	1119	SNV	G	T	56	1428	3,921569	No	CDS:p.Gly301Val	Yes
BEG144_3	1160	SNV	A	G	1389	1409	98,58055	No	CDS:p.Asn315Asp	Yes
BEG144_8	85..86	MNV	CA	AC	78	2728	2,859238	No	CDS:p.Gln29Thr	Yes
BEG144_8	161	SNV	G	A	3493	3526	99,0641	No	CDS:p.Ser54Asn	Yes
BEG144_8	208	SNV	T	A	41	3390	1,20944	No	CDS:p.Tyr70Asn	Yes
BEG144_8	244	SNV	A	C	4193	4201	99,80957	No	CDS:p.Ile82Leu	Yes
BEG144_8	256	SNV	G	A	3336	3949	84,47708	No	CDS:p.Ala86Thr	Yes
BEG144_8	256..257	MNV	GC	CA	60	3949	1,519372	No	CDS:p.Ala86Gln	Yes
BEG144_8	356	SNV	A	G	4765	4793	99,41581	No	CDS:p.Lys119Arg	Yes
BEG144_8	359	SNV	C	T	4716	4718	99,95761	No	CDS:p.Thr120Ile	Yes
BEG144_8	382	SNV	C	T	4125	4162	99,111	No	CDS:p.Leu128Phe	Yes
BEG144_8	718..719	MNV	AT	TA	78	3994	1,952929	No	CDS:p.Ile190Tyr	Yes
BEG144_8	1025	SNV	T	A	4620	4656	99,2268	No	CDS:p.Ser270Thr	Yes
BEG144_8	1119	SNV	G	T	30	1102	2,722323	No	CDS:p.Gly301Val	Yes
BEG144_8	1160	SNV	A	G	930	950	97,89474	No	CDS:p.Asn315Asp	Yes

	Region	Type	Reference	Allele	Count	Coverage	Frequency	Homopolymer	Amino acid change	Non-synonymous
BEG72_1	61	SNV	C	T	2373	2373	100	No	CDS:p.His21Tyr	Yes
BEG72_1	112	SNV	G	A	3670	3676	99,83678	No	CDS:p.Ala38Thr	Yes
BEG72_1	130	SNV	T	C	3822	3855	99,14397	No	CDS:p.Ser44Pro	Yes
BEG72_1	718..719	MNV	AT	TA	99	6051	1,636093	No	CDS:p.Ile190Tyr	Yes
BEG72_1	866	SNV	A	G	7171	7466	96,04875	No	CDS:p.Asn239Ser	Yes
BEG72_1	1119	SNV	G	T	22	1027	2,142162	No	CDS:p.Gly301Val	Yes
BEG72_1	1128..1129	MNV	AC	TA	28	1724	1,62413	No	CDS:p.Asp304Val	Yes
BEG72_1	1163..1164	MNV	CA	AT	821	834	98,44125	No	CDS:p.His316Ile	Yes
BEG72_2	61	SNV	C	T	1117	1117	100	No	CDS:p.His21Tyr	Yes
BEG72_2	112	SNV	G	A	1967	1976	99,54453	No	CDS:p.Ala38Thr	Yes

BEG72_2	130	SNV	T	C	2093	2115	98,95981	No	CDS:p.Ser44Pro	Yes
BEG72_2	220	SNV	C	T	32	2102	1,52236	No	CDS:p.Pro74Ser	Yes
BEG72_2	718	SNV	A	T	38	3617	1,050594	No	CDS:p.Ile190Phe	Yes
BEG72_2	866	SNV	A	G	5160	5387	95,78615	No	CDS:p.Asn239Ser	Yes
BEG72_2	1119	SNV	G	T	8	566	1,413428	No	CDS:p.Gly301Val	Yes
BEG72_2	1163..1164	MNV	CA	AT	406	411	98,78345	No	CDS:p.His316Ile	Yes
BEG72_2	1176	SNV	A	T	3	267	1,123596	No	CDS:p.Lys320Met	Yes
BEG72_2	1178	SNV	A	G	3	196	1,530612	No	CDS:p.Lys321Glu	Yes

BEG72_4	61	SNV	C	T	2083	2084	99,95202	No	CDS:p.His21Tyr	Yes
BEG72_4	112	SNV	G	A	3091	3095	99,87076	No	CDS:p.Ala38Thr	Yes
BEG72_4	130	SNV	T	C	2993	3018	99,17164	No	CDS:p.Ser44Pro	Yes
BEG72_4	718..719	MNV	AT	TA	82	4706	1,742456	No	CDS:p.Ile190Tyr	Yes
BEG72_4	866	SNV	A	G	5714	5960	95,87248	No	CDS:p.Asn239Ser	Yes
BEG72_4	1116	SNV	T	G	12	1157	1,037165	No	CDS:p.Val300Gly	Yes
BEG72_4	1163..1164	MNV	CA	AT	612	617	99,18963	No	CDS:p.His316Ile	Yes
